# Effectiveness of theory-based breast self-examination intervention for breast cancer prevention among female college teachers in Pakistan: A cluster randomized controlled trial study protocol

**DOI:** 10.1371/journal.pone.0321634

**Published:** 2025-04-17

**Authors:** Benazir Mahar, Malina Binti Osman, Fatimah Binti Ahmad Fauzi

**Affiliations:** Department of Community Health, Faculty of Medicine and Health Sciences, University Putra Malaysia, Selangor, Malaysia; Far Eastern University - Manila, Philippines

## Abstract

**Background:**

Breast cancer poses a significant health challenge in Pakistan, with a disproportionately high number of cases diagnosed at advanced stages. Despite the rising incidence, preventative measures like regular screening remain not commonly practiced among Pakistani women. While extensive research exists on breast cancer globally, there is a critical gap in studies specifically designed and evaluated to enhance breast self-examination practices within the Pakistani context.

**Methodology:**

The primary goal of this study is to design and implement an educational intervention on breast self-examination and evaluate its effectiveness among college teachers in Pakistan. This protocol outlines a single-blind, parallel cluster randomized controlled trial (CRCT) with an intervention period of three months. Clusters will be randomly assigned to either the control or intervention groups,and baseline data will be gathered from both groups. An intervention based on the health belief model will be executed for the intervention group to improve women’s knowledge and behaviors about breast self-examination (BSE). Data will be collected at two follow-up intervals for both groups post-intervention. The modified questionnaires include constructs such as breast cancer symptoms, risk factors, detection techniques, frequency and practices of breast self-examination, and perceptions of breast cancer. The control group will get the intervention once the trial concludes. The primary outcome of this study is breast self-examination (BSE) practice, whereas secondary outcomes encompass knowledge and beliefs related to breast cancer and BSE.

**Discussion:**

This cluster randomized controlled trial is aimed to improve the efficacy and legitimacy of theory-based intervention by increasing women’s knowledge of breast self-examination and breast cancer and changing their attitudes to encourage early breast cancer detection by practicing breast self-examination. This might significantly allow an improved detection rate; therefore, earlier treatment can be offered. Therefore, lower the death rate from breast cancer and guide health promotion initiatives in other comparable settings. Furthermore, less aggressive therapies are frequently possible with early detection, which enhances healthcare cost-effectiveness while also improving patient outcomes and treatment burdens.

**Trial registration:**

This study protocol is registered with the Thai Clinical Trial Registry (TCTR), TCTR20240703005 (https://www.thaiclinicaltrials.org/show/TCTR20240703005). The following study protocol complied with the Standard Protocol Items Recommendations for Interventional Trials (SPIRIT) checklist. (S1 file).

## Introduction

Breast cancer accounts for approximately one-third of all new cancer diagnoses in women [1]. Breast cancer (BC) is the most prevalent form of cancer in women globally, posing a serious threat to their health in both developed and developing countries [2].Despite significant efforts and advancements in medical care, breast cancer (BC) is still one of the most serious illnesses for women [3].

The incidence of breast cancer is increasing in emerging countries because of longer life expectancies, increased urbanization, and alterations in lifestyle and reproductive patterns [4]; however, early diagnosis because of early presentation is notably responsible for the recent decline in breast cancer deaths in Western countries [5].

The situation has significantly changed in developing countries; Pakistan’s incidence is lower than that of other Asian countries, but its fatality rate is much greater. Pakistan ranks fourth in Asia in incidence but ranks third in mortality due to breast cancer [6]. Early detection of breast cancer (BC) increases the likelihood of early discovery of BC and successful treatment, saving thousands of lives each year [7]. In Pakistan, particularly among women, the society is not very receptive or open to discussing sexually transmitted diseases or problems related to breast/sexual health [8] The reason why late-stage breast cancer is the common clinical presentation in Pakistan is easily understood [9]. Females in Pakistan in general has lack of awareness about breast cancer and screening methods. Previous studies [10,11] have shown that the main barriers to breast cancer screening for Pakistani women are a lack of awareness about and false health attitudes about breast cancer. A study conducted in one of the major cities in Pakistan reported the same trend only 5.4% of females in Pakistan engage in breast self-examination [12].

Reducing cancer mortality requires preventive measures, and screening as a secondary preventive measure is a wise choice [13]. A necessary predisposing factor for changing behavior is knowledge of the importance of breast cancer screening itself. Improved health-seeking behavior is also a result of knowledge [14]. Moreover, education might significantly alter attitudes, misconceptions, and beliefs, enhancing screening procedures [15]. Several models and ideas have been theoretically employed to understand early BC detection. One such educational intervention to promote awareness is based on the health belief model (HBM), which serves as the theoretical foundation [16]. HBM has demonstrated effectiveness in addressing issues influencing BCS behaviors. Perceived susceptibility,, severity, benefits, barriers, self-efficacy and cues to action are some of the model’s dimensions [17]. According to this paradigm, the person must be persuaded that the sickness or condition may still exist despite the lack of symptoms; consequently, females are more likely to engage in healthy behaviors when they perceive themselves to be at risk for the disease (perceived susceptibility), understand that there may be serious consequences (perceived severity ), think that taking preventive action will have positive results (perceived benefits) and that the benefits could outweigh the risks (barriers) [18].

Although extensive international research has been conducted on breast cancer knowledge, awareness and behaviors of females in the educational sector have received very little attention  when recruiting participants from diverse backgrounds.

In Pakistan, very few cross-sectional studies have been conducted to assess the knowledge and practices of females, and most of these studies were conducted through online surveys [19]. Very little importance is given to interventional studies. Among previously conducted cross-sectional studies, most were among university/college students [20–21], medical staff/nurses in hospitals [22,23] not among females working in the educational sector. Teaching is the highest among all other professions with female employees in Pakistan, and a large number of women practice teaching in this profession, constituting about 60% of the total 1.89 million teachers appointed in private and public educational institutions in Pakistan [24]. In this current research context, studying teachers is important because they are thought to be responsible, knowledgeable, and good sources of knowledge and motivation for their female students. It can be assumed that they can also transfer the extracted information in an effective way in the community. In short, there is a significant knowledge and awareness gap regarding breast cancer and breast self-examination practices among Pakistani women. This study aims to develop, validate, implement, and evaluate the effectiveness of health education intervention grounded in the Health Belief Model to enhance breast self-examination practices among college teachers.

## Methodology

### Ethics and dissemination

#### Research ethics approval and permission.

This study obtained permission and ethical clearance from the College Education Department, Government of Sindh letter no.DCEHRH(INS)2023–24/423, Pakistan, and from the local Institutional Review Committee of Mekan Medical College, reference no. MMC/ERC/1/6/2024.

**Informed consent** Written informed consent will be obtained by the study researcher from all participants who will be willing to the part of the study. An informed consent form is attached as a Supporting file (S3).

### Study design, sample size, study sites, selection criteria, sampling method, and randomization strategy

#### Study time period and design.

**Fig 1:**
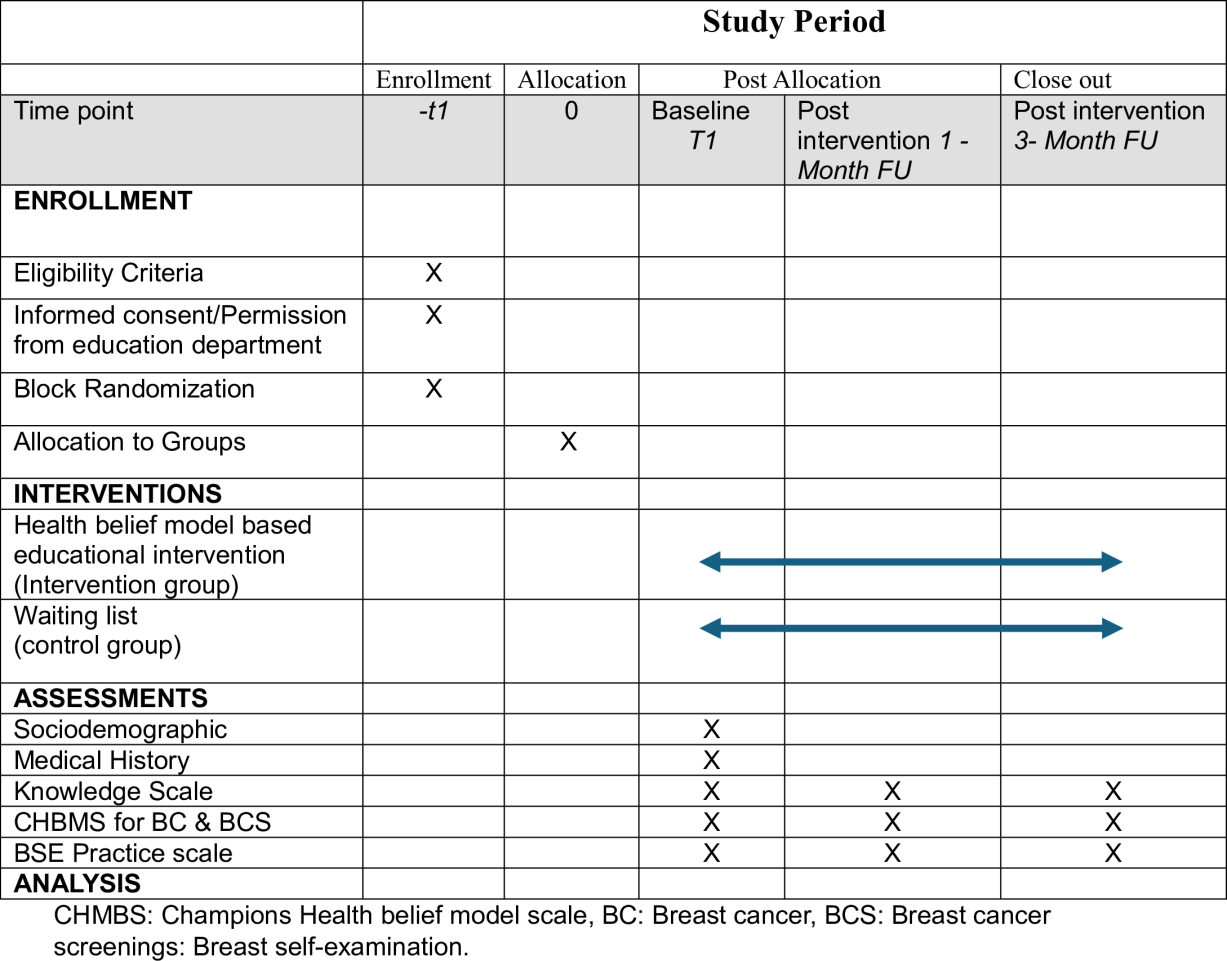
SPIRIT flow diagram of study events.

This study will start recruiting participants for the baseline data collection point on September 4^th^, 2024, and complete data collection for 2^nd^ post-intervention data point on 20^th^ January 2025. The events of this study are illustrated in Fig 1.

**Fig 2:**
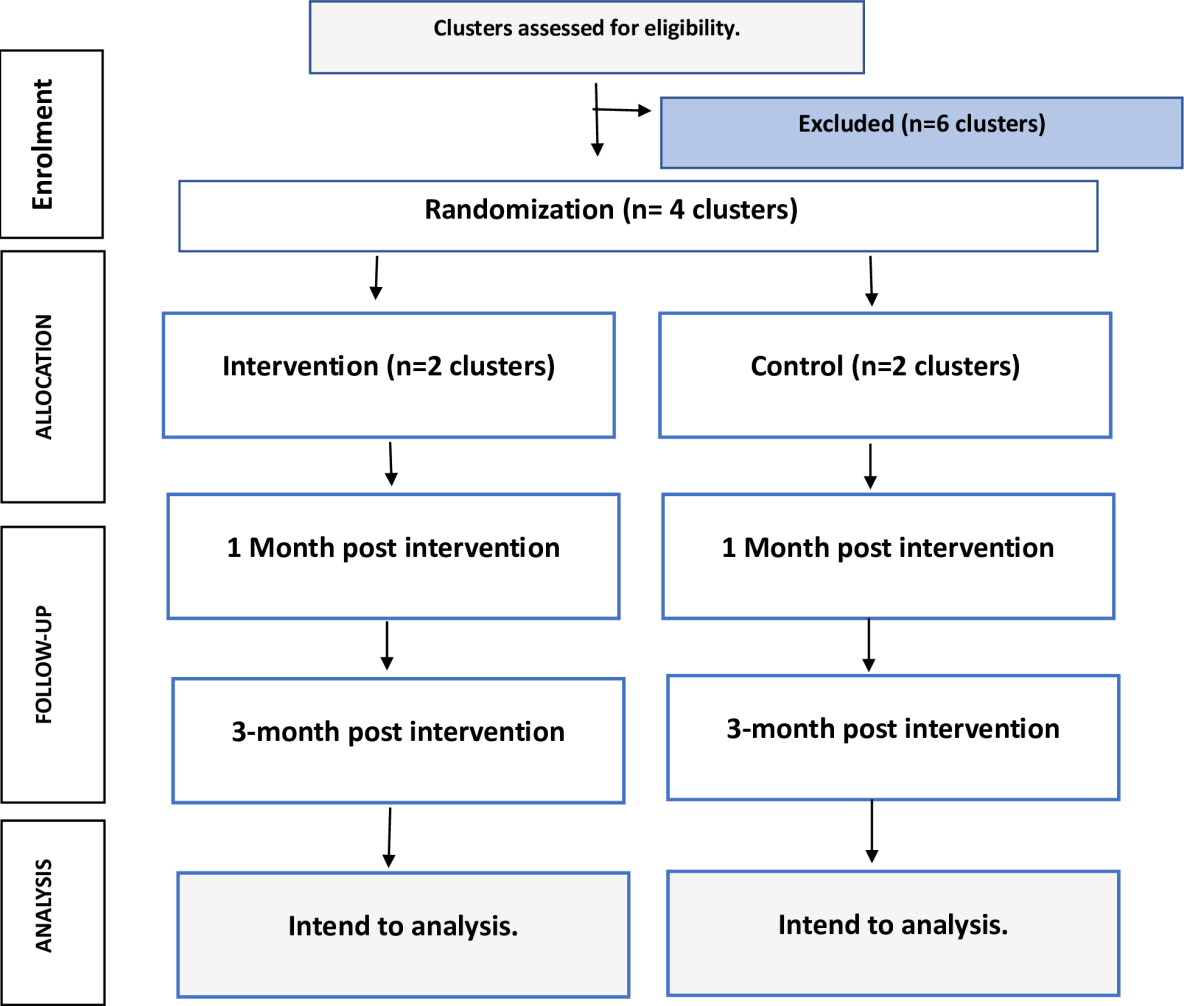
CONSORT flow diagram of the study adapted from Campbell [ 25].

A single-blind, clusterrandomized controlled trial (RCT) will be conducted among female college teachers working in government girls’ colleges in the district of Hyderabad. Blindness is achieved by informing all participants that they are part of a study on breast self-examination without revealing the nature or presence of the intervention in certain colleges. Participants in each college will not be informed whether their college is part of the intervention group or the control group. The colleges will serve as randomization units (clusters). The study will be executed in three phases as shown in Fig 2.

1. **Phase I:** Baseline data collection from both the control and intervention groups.
2. **Phase II:** Delivery of the intervention exclusively to the intervention group.
3. **Phase III:** Postintervention data collection at two time points: one month and three months after the intervention.

The same questionnaire will be used to collect data from both the control and intervention groups at each phase. The key difference lies in the timing of the intervention module: it will be administered to the intervention group during the study, while the control group will receive the same module after the study’s completion.

#### Sample size determination.

The sample size was determined based on a comparison of BSE practice proportions between an intervention and a control group. A baseline BSE practice proportion of 86.5% was established for the intervention group, while 26% was used for the control group, as per the literature [26] The statistical testing method used to achieve 80% power in this sample size calculation is a two-sample z-test for the difference between two proportions. Considering, a significance level of 0.05, and a design effect of 1.9 to account for clustering, a sample size of 42 participants per group was calculated. To accommodate potential attrition (20%) and eligibility criteria (90%), the final sample size was adjusted to 57 participants per group.

#### Study setting.

This study will take place in the Hyderabad region located in Sindh Province. The study sites will be the government colleges for girls located in the Hyderabad region.

#### Eligibility criteria for the selection of clusters and participants.

The criteria for including clusters were Government Girls’ colleges located in Hyderabad willing to participate in the study and respondents who were appointed to government colleges in Hyderabad district, aged between 25–59 years and willing to participate in the study.

The criteria for excluding clusters were that colleges that refused to participate in the trial, that they had already participated in a study conducted for the pretesting of questionnaires, pilot studies, and cross-sectional studies conducted before this CRCT, and that the exclusion criteria for participants were breast cancer patients/survivors, pregnant and lactating mothers, and females who did not like to participate or who were on sabbatical leave during the data collection period.

**Confidentiality**: To ensure the confidentiality of participant information, we will implement secure measures before, during, and after the trial. Each participant will be assigned a unique code to separate identifiers from research data. Only authorized study researchers will have access to this information, and data will be shared only in anonymized form.

#### Sampling method.

**Fig 3:**
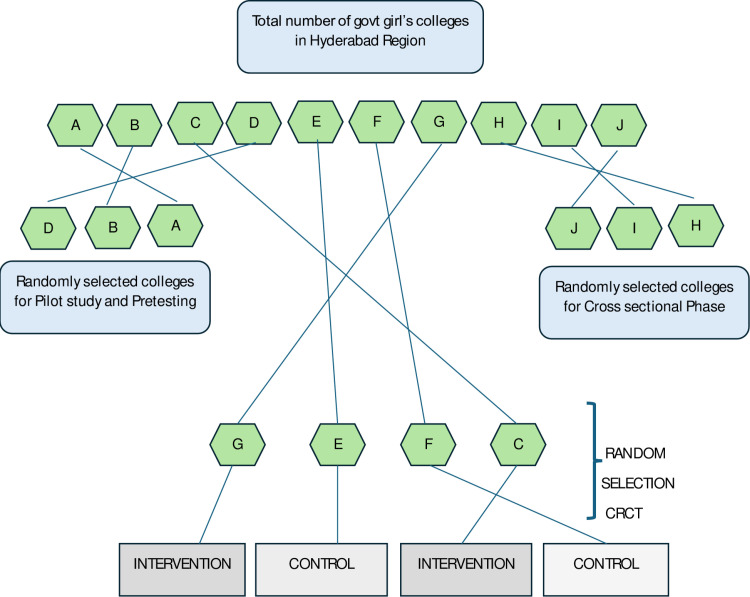
Total number of eligible clusters in the Hyderabad region and their selection strategy.

This study’s sampling strategy comprised many carefully planned stages to ensure a representative and balanced sample of college teachers. The initial step is an assessment of eligibility criteria, in which colleges will be assessed on factors such as the necessary infrastructure, required number of teachers, and consent to participate. This step is important to ensure that included colleges are appropriate, which increases the reliability of the study. There are a total of ten eligible colleges in Hyderabad; three of them were randomly selected for the pilot and pretesting of instruments. Later, three more colleges were randomly selected for the cross-sectional phase, and finally, four colleges were left to include in the main study CRCT phase, as shown in Fig 3.

**Fig 4:**
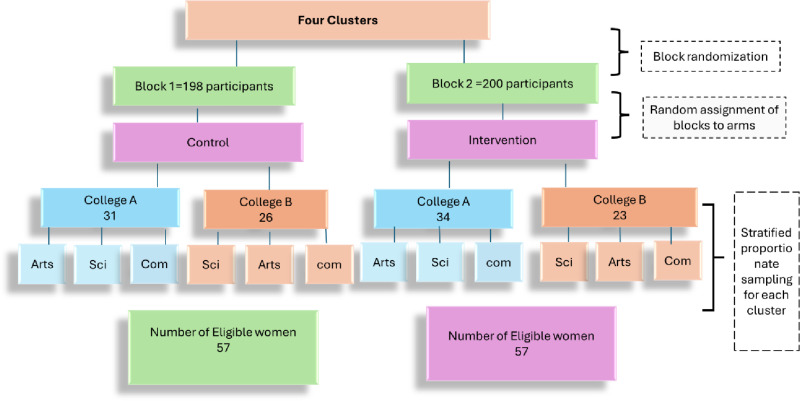
Sampling procedure of the study participants.

The next stage is the blocking of colleges, in which included colleges are grouped into two blocks almost balanced in size, based on the total number of teachers. This strategy is used to ensure comparability between the intervention and control groups by achieving balance and minimizing possible biases that may result from variations in college size. The blocks of our study included Block 1: 200 teachers (College A=112, College B=88) and Block 2: 198 teachers (College A=117, College B=81). The third stage is” randomization” within blocks. Using the flipping-a-coin approach, each block is randomly allocated to either the intervention group or the control group. This step is included to ensure that the allocation to treatment groups is completely by chance, thus minimizing selection bias. Given that the researchers are aware of the group allocation (intervention and control colleges) but the participants are not, the study employs a single-blind design. The next step is the proportionate distribution of sample size, where the target sample size is distributed among the colleges in accordance with the number of faculty members at each college. This is a crucial step in ensuring that every college participates equitably in the sample size and that the study remains balanced. The sample is then stratified by faculty (arts, science, and commerce/ business) within each college within the proportionate stratified sampling phase. The required number of participants from each faculty is calculated proportionally. This stratification is critical to ensure adequate representation of all faculties in the sample, which improves the generalizability of research findings. Lastly, individuals within each stratum are chosen at random using a random number generator during the random sampling step. After discussing the inclusion and exclusion criteria and obtaining written consent, the individuals selected will be asked to participate in the study. Those who provide their assent will have their baseline data gathered. This step maintains ethical norms and improves the quality of the data gathered by guaranteeing an impartial selection procedure and complete participant disclosure. By going through these steps, the study intends to obtain a representative and balanced sample, which will improve the overall validity and reliability of the research findings, Fig 4).

## Data collection and management

### Recruitment of participants

Followed by proportional stratified sampling participants will be randomly selected from each stratum using a random number generator from the list provided by the education department. Selected participants will be invited to participate in the study. The researcher provides a brief overview of the study and explains its benefits and potential risks to those who meet the participation criteria. Those who meet the criteria will be asked for written informed consent. After completing the consent forms, participants can withdraw from the study at any time without penalty or loss of benefits and are not asked to provide a reason in advance. Finally, baseline data will be collected from the participants who provide consent.

### Intervention and follow-up

After baseline data collection, colleges assigned to the intervention group will be exposed to the educational intervention. After the intervention, the teachers who were randomly selected in intervention and control groups will be contacted for a follow-up evaluation. These follow-ups will be conducted one month and three months after the intervention to assess the ongoing impact of the training program on participants’ knowledge, beliefs, and practices. Follow-up assessment involves collecting data on the same variables measured at baseline, which allows for comparisons of changes over time. A systematic follow-up process ensures that studies can accurately track the effectiveness of an intervention and provide insight into its long-term benefits.

### Plans for retention and follow-up of participants

Participants will be strongly encouraged to attend both the intervention and post-intervention follow-up sessions. However, participants retain the absolute right to withdraw from the study at any point without providing a reason. To minimize attrition, various strategies will be implemented to foster participant retention. Participants will be asked to provide their contact information to facilitate communication and potential creation of a study-related group. All participant information will be handled with strict confidentiality.

### Data management

Upon joining the study, each participant receives a unique study ID. Participants will be identified by this number in all study-related documents during the intervention and data analysis. All study data are properly protected to meet administrative requirements related to the collection of personal data.

## Interventions, study outcomes analysis plan and study instrument.

### Intervention

The TIDieR checklist was used to describe the elements of the intervention [27]. The TIDieR (Template for Intervention Description and Replication) checklist is designed to help research projects report more thoroughly, especially when it comes to effectively describing treatments. By using this checklist, the intervention is reported in a way that makes it possible for other researchers to replicate it. (S2 Checklist).

This study implements a program titled “Breast Cancer Awareness and BSE Practices Educational Intervention Trial.” The intervention, grounded in the Health Belief Model (HBM), aims to improve knowledge and self-reported practices of breast self-examination (BSE) among female college teachers. The HBM framework suggests that individuals are more likely to adopt preventive health behaviors when they perceive a susceptibility to a health threat (breast cancer in this case), believe in the seriousness of the condition, and have confidence in the effectiveness of the recommended behavior (BSE for early detection).

The program is designed based on educational materials and skills training, aiming to address these core HBM constructs. The development involved a review of existing educational resources and consultation with college staff to ensure feasibility and content appropriateness. The intervention consists of several components delivered to the intervention group:

A one-day educational program on breast self-examination (BSE) will be held for a total of 57 teachers across two colleges . A focused one-hour session will be held at each college, facilitated by a qualified nursing assistant and a study researcher. The assistant will lead participants through a practical training session to make sure they understand the correct method, while the researcher will present multimedia on breast cancer and the significance of BSE. The researcher will visit both colleges in advance to verify that the required multimedia materials are available to guarantee a seamless presentation.

An educational session lasting an hour will cover topics such as breast anatomy, cancer awareness, screening strategies, and a brief film on appropriate BSE techniques. The risk factors for breast cancer, the possible repercussions of a delayed diagnosis, and the advantages of early identification by BSE will all be highlighted in this section. A skills training session using a breast model to demonstrate palpation techniques and signs of concern. This will enhance participants’ confidence in performing BSE effectively. The take-home materials included a leaflet summarizing key points, a short educational film on BSE sent via WhatsApp, a wall hanging, a writing pad, pen, and keychain with a breast cancer awareness logo, and monthly BSE reminder messages delivered through WhatsApp.These materials serve as ongoing prompts and resources, reinforcing the importance of BSE. Souvenirs with a breast cancer awareness message serve as reminders for regular BSE practice.

**Fig 5:**
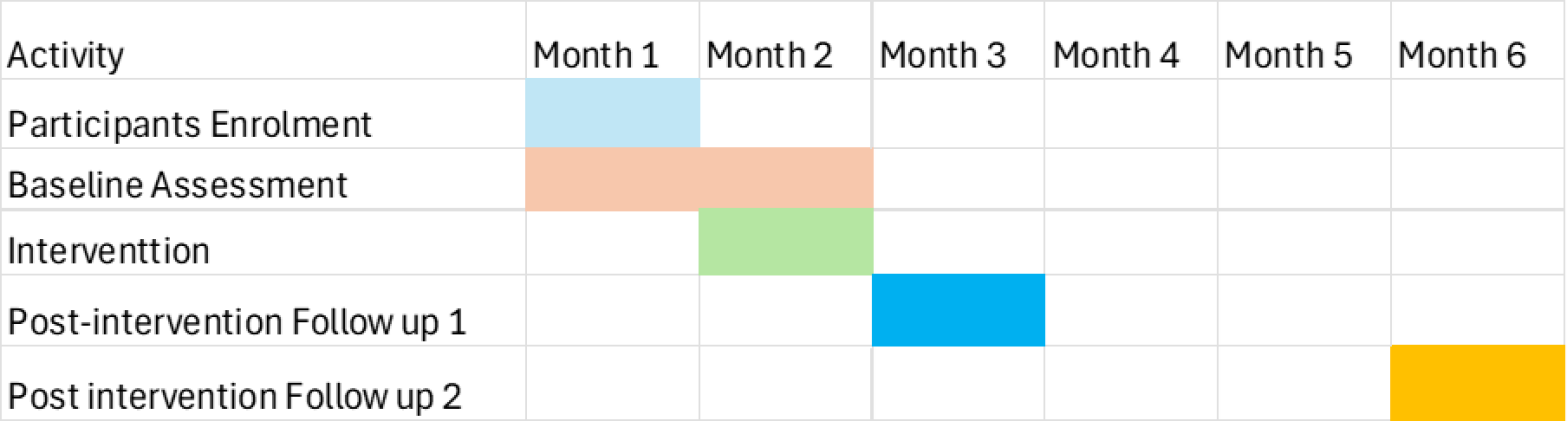
Timeline of enrolment, intervention, and assessment of study participants.

The control group will receive the same educational materials and training after the final data collection from both groups. The timeline of enrolment, intervention, and assessment is provided in Fig 5.

### Intervention validity

Several strategies will be used to support the validity of educational interventions. They include a strategy to maintain the continuity of the intervention, motivate participants to commit to the intervention, and strengthen their commitment. To maintain intervention consistency,

The same researcher implements the educational intervention for each college.

The researcher undertakes to implement a uniform training intervention protocol to ensure standardized delivery in both intervention colleges. The BSE training is designed and delivered by an expert nursing assistant. The researcher observes the sessions and provides feedback to the study participants wherever required. Additional approaches used to enhance fidelity will be sending brief reminders to participants and their WhatsApp groups monthly, as well as providing merchandise featuring the BC logo, such as pens, keychains, writing pads, and stickers. They are primarily used to encourage participants and act as a reminder for them to practice BSE regularly.

## Statistical analysis

### Baseline measurements

To evaluate the gathered data, IBM SPSS Software 29.0 will be used. Data capturing will be performed using Microsoft Excel, which allows for efficient entry and organization of data before analysis in SPSS. At baseline, participants’ characteristics and study variables of both arms will be measured, and the clusters will be compared at the baseline level for any differences among their characteristics, knowledge level, beliefs and practices. The normality of continuous variables will be checked before analysis. A value of 0.05 will be chosen as the alpha level of significance. The data at baseline will be described using descriptive analysis., The chi-square test will be used to examine the differences in observed proportions of categorical data between the intervention and control groups, when comparing within-group comparisons, in a 2×2 table including a cell with an anticipated count below 5,Fisher’s exact test will be employed to assess the association between the two groups To compare the mean difference in continuous data between the intervention group and the control group, the independent-sample t-test will be used between normally distributed variables, where as the Mann-Whitney U test will be employed to compare continuous data that are not normally distributed.

### Postintervention measurements

At the end of the study, the pre- and post-intervention scores of the participants will be compared within the groups and between groups. Cochran’s Q test will be used to assess changes in the proportions of categorical data over time within the study groups. This analysis will determine whether there are significant differences in the proportion of successes between the intervention and control groups. To determine the mean difference for continuous variables across time within the study groups, one-way repeated-measures ANOVA will be used.

Lastly, a generalized estimating equation (GEE) will be performed to assess overall impact of the intervention, The GEE is a robust and widely used type of analysis employed for analysing clustered, longitudinal data. It will help to assess the overall impacts of group, time, and group–time interaction effects on the primary and secondary outcome variables. Additionally, baseline covariates demonstrating significant differences between the intervention and control groups will be adjusted during analysis. The confidence interval (CI) for mean estimations is set at 95%, and the significance threshold, alpha (α), is set at 0.05. Intention-to-treat analysis, which accounts for any loss to follow-up or dropout instances, will be utilized in comparison to per-protocol analysis.

### Intention-to-treat strategy

The ITT strategy will be used in the follow-up data analysis. To incorporate an ITT strategy, all participants randomized to the intervention and control groups will be included in the follow-up data analysis, regardless of their participation or dropout. If a participant does not complete the follow-up assessment, their last available data point is used to impute missing data. This method called the last observation carried forward (LOCF)[28], helps maintain the integrity of the ITT analysis and reduces the potential bias of missing data.

### Study measures

The current study will include one primary outcome and two secondary outcomes, which are represented in Table I.

**Table 1:** Primary and secondary outcomes of the study.

Outcome	Elements				
	Domain	Specific Measurement	Specific Metric	Method of aggregation	Time points
**Primary**					
BSE practice	Percentage of participants consistently and proficiently doing BSE	BSE Practice	Self-report answer instrument on BSE practice and performance frequency	Regular (once a month) or irregular (other responses)	Baseline 1 month 3 months
**Secondary**					
Knowledge of breast cancer and BSE	Knowledge of breast cancer, risk factors, signs and symptoms, and screening.	Knowledge of Breast Cancer and BSE	Self-report questionnaire on breast cancer and BSE	Total knowledge score (out of the 36 as maximum score)	Baseline 1 month 3 months
Beliefs about breast cancer and BSE	Perceived severity, seriousness, benefits, barriers, confidence, motivation.	Beliefs about Breast Cancer and BSE	Modified Champion’s Health Belief Model Scale	Total belief score (out of 160 as maximum score)	Baseline 1 month 3 months

### Primary outcome

**BSE** **practices:** The percentage of participants who consistently and proficiently performed breast self-examinations. A self-report answer instrument, which consists of the following questions on breast self-examination, will be used to gauge BSE practice and performance frequency. Any other response will be labeled nonpracticing (irregular BSE), while a woman who performs BSE once a month will be regarded as practicing (regular BSE).

### Secondary outcome

**Knowledge of breast cancer and BSE:** Knowledge of breast cancer, risk factors, sign symptoms, and knowledge associated with breast self-examination will be measured by using a self-report questionnaire. The questionnaires consisted of 36 items. The questions asked about breast cancer risk factors, signs and symptoms, breast health awareness (BSE), and breast cancer screening techniques. Beliefs about breast cancer and BSE. The modified Urdu version of the Champions Health Belief Model Scale (CHBMS), which was developed by V. Lee Champion and translated and validated by the researchers of this study, will be utilized to measure the beliefs of participants. The scale comprises 32 questions divided into six subscales covering all six health domains: perceived severity and seriousness of breast cancer, benefits and barriers of breast self-examination, self-efficacy in performing self-examination, and cues to action.

#### Validity and reliability ofstudy instruments

The questionnaire used for this cRCT is a validated and reliable tool; the validity and reliability of this instrument are tested by the study researchers before conducting the current controlled trial. The Cronbach alpha value for all 6 belief constructs of the health belief model ranged between 0.8–0.9.

The data-collecting tool for this study is generally tailored to the study’s context and derived from a variety of previous research, which enhances the measures’ reliability. The reliability is evaluated with Cronbach’s alpha, and necessary corrections are made based on the test results. The cut-off value used for the Cronbach’s alpha test is 0.70 or higher. Additionally, the same data collectors will be employed to evaluate research participants at baseline and after the survey as an additional measure to guarantee the instrument’s reliability.

#### Plans for results dissemination

Best-practice recommendations will guide the dissemination process. The goal is to disseminate the results of the present study to teachers, young females, public health experts, and policyholders. To reach academic and clinical audiences, the findings will be presented at national and international conferences and published in peer-reviewed publications.

#### Authorship

The authorship will be done in accordance with the guidelines provided by the International Committee of Medical Journal Editors (ICMJE).

## Discussion

The goal of this study is to investigate the effects of a theory-based intervention to increase breast self-examination knowledge and the intention to promote health behavior related to the timely screening of breast cancer patients. To the best of our knowledge, this study is the first interventional study at the community level to assess and create a strategy for promoting breast self-examination practices in Pakistan. At the completion of the current project, we will rigorously evaluate the effectiveness of a novel intervention addressing breast cancer awareness about symptoms and risk factors and improving knowledge, beliefs, and regular practices related to breast self-examination, particularly timely screening.

Patients and their families are thought to bear particularly heavy pressure because of late-stage BC [29]. Additionally, it places financial constraints on healthcare systems, communities, and economies [30].By including an under-represented community, our educational program acts as a strategy for BC prevention.

The goal is to improve BCS results and lessen inequalities. This study aimed to support the efficacy and legitimacy of using a theory-based intervention designed to inform women about BCS The anticipated results provide a deeper understanding of the role of knowledge and beliefs in encouraging BCS adoption. This research not only immediately benefits educators, their pupils, families, and friends but also educates researchers and healthcare professionals who seek to develop interventions for women.

The information from this study could be used to design suitable BCS programs for other women in various settings. To disseminate relevant information extensively throughout communities, these programs boost health systems. They could be used to ensure that specific groups are known at both the individual and society levels.

To the best of the researcher’s knowledge, this study is the first to implement breast self-examination educational intervention among teachers using a theoretical framework. The effectiveness of this educational intervention will be assessed, and it will be put into practice in a variety of venues where health education interventions for women are offered.

## Conclusion

The current study aims to bridge a significant gap in knowledge by assessing the efficacy of a theory-based intervention designed to increase breast self-examination practices among Pakistani teachers. By targeting a previously unexplored population and employing a rigorous methodology, this research contributes valuable insights into breast cancer prevention strategies. Successful implementation of the intervention holds the potential to improve breast cancer outcomes through early detection, reduce health disparities, and inform the development of tailored interventions for diverse populations. The findings of this study are expected to have far-reaching implications for public health policies, educational programs, and future research endeavors in the field of breast cancer prevention.

## Supporting information

S1 File SPIRIT checklist. (DOCX)

S2 File TIDieR checklist. (DOCX)

S3 File Informed consent form. (PDF)

S4 File Protocol. (DOCX)
