# Supplementary material for: Effectiveness of theory-based breast self-examination intervention for breast cancer prevention among female college teachers in Pakistan: A cluster randomized controlled trial study protocol
Source: PLoS One. 2025 Apr 17;20(4):e0321634. doi: 10.1371/journal.pone.0321634 (PMC12005520; doi:10.1371/journal.pone.0321634)
Supplement: S3 File — (PDF) [file pone.0321634.s003.pdf]

RESPONDENT'S INFORMATION SHEET AND CONSENT

UNIVERSITI PUTRA MALAYSIA,
43400 UPM SERDANG, SELANGOR, MALAYSIA

I. STUDY TITLE

Effectiveness of a health belief model-based educational intervention on knowledge, beliefs, and practices of breast self-examination among college teachers in Hyderabad, Pakistan.

II. INTRODUCTION

Breast cancer is the most frequently occurring cancer type among females worldwide. Pakistan is one of the Asian countries with a higher prevalence and immature mortality due to breast cancer and ranks top among all the other cancer types in prevalence and death. In Pakistan, 82% of patients are diagnosed late and among them, 52% are diagnosed at an advanced stage with very less or almost no survival chances. Breast cancer greatly impacts the physiological and psychological well-being of patients; furthermore, available cancer treatment also exerts severe health consequences, emotionally and physically. However effective population-based screening strategies can make it possible to detect cancer at the very initial stage among asymptomatic females. Thereby reducing the burden of disease both for patients and for the health-providing system. Breast self-examination is the technique by utilizing that females can detect any abnormal tumor in their breast at home without the assistance of any health care provider or any equipment. Breast self-examination is a self-awareness strategy that assists individuals in recognizing the normal structure and shape of their breasts. It empowers them to promptly identify any abnormal signs or symptoms associated with breast cancer. Breast self-examination is generally practiced by females in developed countries and included in their routine however in Pakistan earlier research reported that it is very less practiced by Pakistani females and

intervention and awareness sessions are recommended by early researchers to improve BSE practices. The current study aims to determine the impact of a health belief model-based educational intervention on knowledge, beliefs, and practices of breast self-examination among college teachers in Hyderabad, Pakistan.

III. WHAT WILL YOU HAVE TO DO?

You are required to only sign the informed consent form to show your willingness to be part of this study. If you are willing to participate in the current study, your cooperation is required to:

i) Fill out the questionnaire at three-time points.

ii) This questionnaire is composed of four sections (Socio-demographic characteristics, risk factors of breast cancer, knowledge and beliefs about breast cancer, and BSE practices).

IV. WHO SHOULD NOT PARTICIPATE IN STUDY?

Teachers who have been diagnosed with breast cancer. Teachers who are absent on the day of intervention and who are planning to go on long leave for the next three months and teachers who are retiring during the study period cannot take part in the study.

V. WHAT WILL BE THE BENEFITS OF THE STUDY?

a) TO YOU AS THE SUBJECT

As a subject, the current interventional study will make you more knowledgeable about breast cancer and develop stronger opinions regarding its early diagnosis. The study's findings may change how you personally feel about breast cancer screening and inspire you to conduct breast self-examination in the right way and at the right time.

b) TO THE INVESTIGATOR

Your involvement in this study will benefit the field of community health by allowing for the provision of better information, safety measures, and preventative measures to teach women about breast cancer early detection. Health educators may be helped by the provision of baseline data on the degree of breast cancer awareness among Pakistani women in promoting prevention programs.

VI. WHAT ARE THE POSSIBLE RISKS?

It is expected that there will be no risk of any kind to study participants and organizations while conducting this study.

VII. WILL THE INFORMATION THAT YOU PROVIDE AND YOUR IDENTITY REMAIN CONFIDENTIAL?

Results of current research will be reported collectively, without any kind of reference of the college or participants of the study, hence the details of participants will be kept confidential, and results will be used for only research purposes.

VIII. WHO SHOULD YOU CONTACT IF YOU HAVE ADDITIONAL QUESTIONS DURING THE RESEARCH?

If you have any questions related to the study, please contact:

**Research Student**	**Main Investigator**
Ms. Benazir Mahar	Dr. Malina binti Osman
Department of Community Health	Department of Community Health
Universiti Putra Malaysia	Universiti Putra Malaysia.
Email: gs65676@student.upm.edu.my	Email: malinaosman@upm.edu.my
Tel: +92331-3061210	Tel: +60136298572

Please sign here if you have read and understood the contents of this page.______

1. CONSENT

I …………………………………… Identity Card No. ……………………………

address………………………………………………………………………………

…………………....……………………………………….............……… I hereby freely consent to participate in the research described above (clinical/drug trial/video recording/focus group/questionnaire-based interview).

According to the Respondent's Information Sheet, I have been informed about the research's methodology, potential drawbacks, and issues. I am aware that I have the option to leave this research at any moment and without providing any explanation. I know that the information I disclose regarding my identity will be kept private and secret, and this study is confidential.

Signature …….………………………… Signature …….…………………………….

(Respondent) (Witness)

Date ....

Name………………………….

I/C No. ………………………………….

I confirm that I have explained to the respondent the nature and purpose of the above-mentioned research.

Date …….…………………………… Signature …….………………………….

(Researcher)
