# Supplementary material for: Effectiveness of theory-based breast self-examination intervention for breast cancer prevention among female college teachers in Pakistan: A cluster randomized controlled trial study protocol
Source: PLoS One. 2025 Apr 17;20(4):e0321634. doi: 10.1371/journal.pone.0321634 (PMC12005520; doi:10.1371/journal.pone.0321634)
Supplement: S4 File — (DOCX) [file pone.0321634.s004.docx]

“**EFFECTIVENESS OF A** **HEALTH BELIEF MODEL-BASED EDUCATIONAL INTERVENTION ON KNOWLEDGE, BELIEFS, AND PRACTICES OF BREAST SELF-EXAMINATION AMONG COLLEGE TEACHERS IN HYDERABAD, PAKISTAN"**

**Approved by the Ethical review committee of Mekran Medical College, Turbat, Pakistan**

**Letter ref.no. MMC/ERC/1/6/2024**

**Protocol date & Version: Date:1: 1st April 2024**

**Principal Investigator: Dr. Malina Binti Osman**

**Co-investigator: Benazir Mahar (Ph.D. Scholar)**

## Study Summary

| **Title** | **The Impact of Educational Intervention on Knowledge, Beliefs, and Practices of Breast Self-Examination among College Teachers: Protocol for a Cluster Randomized Control Trial** |
| --- | --- |
| **Background** | Breast cancer is a significant health concern, becoming more likely for women over time. Despite numerous studies on this topic among women in many countries, few have examined the importance of breast cancer screening among Pakistani females. This study aims to design, implement, and evaluate educational interventions for breast cancer prevention targeting female college professors in Pakistan. |
| **Primary objective** | To design, implement, and evaluate educational interventions for breast cancer prevention targeting female college professors in Pakistan. |
| **Methodology** | This study protocol is based on a single blind, parallel cluster randomized controlled trial (CRCT) design with an intervention time of 3 months. The clusters will be randomized into control and treatment groups, and baseline data from both groups will be acquired. A health belief theory-based breast self-examination intervention will be delivered to the intervention group, followed by the collection of post-intervention data at two different time points from both groups. The questionnaires will include basic questions on breast cancer symptoms, risk factors involved, detection methods, practices and frequency of performing breast self-examination, and beliefs about breast cancer. The control group will receive the intervention at the end of the study. |
| **Study Design** | Cluster randomized controlled trial (CRCT) |
| **Eligibility Criteria** | A. Inclusion criteria  For colleges  Willing to be a part of the study.  • For teachers  • Full-time faculty appointed in government colleges in Hyderabad district.  • Aged between 25-59 years.  • Willing to be part of the study.  B. Exclusion criteria  • For colleges  • Colleges that are not willing to participate in the study despite meeting the inclusion criteria.  • Colleges already participated in initial phases of study, pilot testing, and cross-sectional study.  • For Teachers  • Pregnant teachers and lactating mothers  • Those who are on sabbatical leave.  • Diagnosed/Recovered breast cancer patients.  • Teachers who will refuse to be part of the study |
| **Sample size** | 114 for both arms, 57 in each arm. |
| **Intervention** | Health belief theory-based breast self-examination intervention delivered to the intervention group |
| **Control Group** | Will receive the intervention at the end of the study |
| **Data Collection Points** | Baseline, post-intervention at two different time points, 1 month and 3 months. |
| **Primary Outcome** | Practice and frequency associated with breast self-examination |
| **Secondary Outcome** | Knowledge of breast cancer and breast self-examination, beliefs linked with breast cancer and breast self-examination |
| **Discussion** | This cluster randomized controlled trial aims to improve the efficacy and legitimacy of theory-based breast self-examination interventions by increasing women's knowledge of the need for breast cancer awareness and changing their attitudes to encourage early breast cancer detection. |

| Chief Investigator &Main supervisor | Dr. Malina Binti Osman |
| --- | --- |
| Co-Investigator &Co-supervisor | Fatimah Ahmad Fauzi |
| Co-Investigator | Benazir Mahar |

**Table of Contents**

[Study Summary 2](#_Toc172828013)

[Introduction 6](#_Toc172828014)

[1.1 Rationale and Background of the study 6](#_Toc172828015)

[1.2 Research Objectives 7](#_Toc172828016)

[1.3 Theoretical framework based on Health belief model. 9](#_Toc172828017)

[1.4 Application of Champion’s Health Belief Model on breast cancer screening 10](#_Toc172828018)

[1.5 Conceptual framework of study 11](#_Toc172828019)

[Methodology 13](#_Toc172828020)

[2.1 Study Location 13](#_Toc172828021)

[2.2 Study Design 13](#_Toc172828022)

[2.3 Study Population 13](#_Toc172828023)

[2.4 Study Duration 13](#_Toc172828024)

[2.5 Sampling Strategy 14](#_Toc172828025)

[2.5.1 Sample Population 14](#_Toc172828026)

[A. Inclusion Criteria: 14](#_Toc172828027)

[B. Exclusion Criteria: 14](#_Toc172828028)

[2.5.2 Sampling Frame 14](#_Toc172828029)

[2.5.3 Sampling Unit 15](#_Toc172828030)

[2.6 Sample Size Calculation 15](#_Toc172828031)

[2.7 Sampling Methods 15](#_Toc172828032)

[2.8 Study outcomes 18](#_Toc172828033)

[2.9 Study instrument 20](#_Toc172828034)

[2.10 Quality control of the questionnaire 22](#_Toc172828035)

[2.11 Data collection 25](#_Toc172828036)

[2.12 Delivery of intervention module and participation 25](#_Toc172828037)

[2.13 Retention of Participants 27](#_Toc172828038)

[2.14 Evaluation of the Educational Intervention 27](#_Toc172828039)

[2.15 Development and validation of intervention module 28](#_Toc172828040)

[2.16 Data summarizing and Analysis 30](#_Toc172828041)

[2.17 Results dissemination 30](#_Toc172828042)

[2.18 Permissions and Ethical clearance 31](#_Toc172828043)

[References 31](#_Toc172828044)

[Appendix I. Study Gantt Chart 33](#_Toc172828045)

[Appendix II. Sample size calculations 34](#_Toc172828046)

[Appendix III. RESPONDENT’S INFORMATION SHEET AND CONSENT & INSTRUMENT 36](#_Toc172828047)

# Introduction

## 1.1 Rationale and Background of the study

Breast cancer ranks second globally among all cancers, with 1.7 million cases (11.9%), yet it ranks fifth in terms of mortality (522,000, 6.4%) due to its relatively favorable prognosis (Fahad Ullah, 2019) It is the most prevalent cancer in women worldwide, affecting both developed and underdeveloped regions, with higher incidences in less developed areas (883,000) compared to more developed ones (794,000)(Ramdas, Benn, & van Heerden, 2020). Pakistan stands among the top Asian countries in terms of breast cancer cases, affecting one in every nine women.

Breast health awareness is critical for early detection. Breast self-examination (BSE) is recommended monthly by the WHO to help women become familiar with their breast tissue and detect any changes early. However, BSE is significantly underutilized in Pakistan due to low awareness and knowledge about the technique(Javaeed, Rasheed, & Shafqat, 2021).This lack of awareness contributes to delayed diagnoses, with 89% of Pakistani patients diagnosed late, often at advanced stages(Saeed, Asim, & Sohail, 2021). Social and cultural attitudes towards breast cancer play a significant role in the delayed presentation, influenced by factors such as age, lack of awareness, and fear of treatment (Agha & Rind, 2021; Sobri et al., 2021).

While breast screening is crucial for detecting asymptomatic lumps early, the practice remains uncommon in Pakistan, further highlighting the need for increased awareness and education(Fitzgerald et al., 2022; Miller et al., 2020). Breast self-examination offers a straightforward method for women to monitor their breast health regularly and detect changes promptly(Oguta et al., 2022).

Despite the importance of BSE, many Pakistani women, including those in professions such as teaching, lack adequate knowledge and practice of this self-examination method (Shahani et al., 2020). Teaching is predominantly a female profession in Pakistan, with women comprising 60% of the 1.89 million teachers across private and public institutions(Government of pakistan 2022).Recognizing teachers as influential advocates for health behaviors among students, educational interventions targeting this group can effectively promote BSE and enhance breast health awareness at a societal level.

In conclusion, leveraging the role of teachers in promoting BSE among students and peers through targeted educational interventions is crucial. Such initiatives not only empower women with essential health knowledge but also contribute to improving early detection and reducing breast cancer mortality in Pakistan.

## 1.2 Research Objectives

To develop, validate, implement, and evaluate the effectiveness of a health educational intervention grounded on the health belief model in improving breast self-examination practices among college teachers.

**Specific Objectives**

1. To develop, validate, implement intervention, and evaluate the effectiveness of a health educational intervention in improving knowledge of breast cancer, knowledge, beliefs, and practices of breast self-examination.
2. To determine a significant association between the respondent’s characteristics, knowledge of breast cancer, knowledge, beliefs, and practices on breast self-examination among college teachers.
3. To determine the differences between and within the control, and intervention group, pre and post intervention on knowledge of breast cancer, knowledge, beliefs, and practice of breast self-examination at baseline, 1 month, and 3 months post interventions.

## 1.3 Theoretical framework based on Health belief model.

The HBM is centered on individuals' perceptions of their vulnerability to and seriousness of a disease, prompting them to take proactive steps to prevent it (Hayden, 2022).When individuals understand the risks and consequences of a disease and perceive themselves as susceptible, they are more likely to adopt preventive measures. Additionally, the model posits that individuals are inclined to engage in preventive practices if they believe that the benefits outweigh any psychological or financial costs associated with prevention (Ralph.J.Clemente, 2021).By targeting beliefs and attitudes, the HBM aims to interpret and predict health behaviors, guiding individuals toward adopting recommended medical advice and preventive measures(Almutari & Orji, 2021).

In essence, the HBM underscores the importance of individuals' perception of disease threat and consequences in influencing their health behaviors, thereby facilitating proactive health practices such as regular BSE for early detection of breast cancer(Htay et al., 2021).

**Fig** I Theoretical framework of the interrelation of HBM's concepts and relationship to behavior change. Source:(K. Glanz, Rimer, & Viswanath, 2008)

## 1.4 Application of Champion’s Health Belief Model on breast cancer screening

Evaluations of the BCS-related HBM constructs have been connected to both BSE and MMG behaviors (Champion, 1993, 1999). In the beginning, Champion created and approved scales for perceived susceptibility in 1984. BSE's perceived benefits, perceived advantages, and perceived barriers. Later,

in 1993, these assessments underwent adjustment, and a self-efficacy scale measure for BSE was introduced. The advantages and challenges of MMG were later updated(Champion, 1999).

The perception of benefits from BSE and MMG was used to address the advantages of screening behavior (BSE or MMG) to reduce the likelihood of death from BC. Finding a mass early, when BC can be treated, was one benefit. Barriers included modules like the anxiety of discovering a mass, the length of the exam, and the pain and radiation associated with the MMG test. Champion and colleagues also created a mammography-related self-efficacy scale. In which ten elements best describe a woman's confidence in her ability to follow the processes required to have an MMG screening(Champion, Skinner, & Menon, 2005).

The initial adaptation and validation of the Champion HBM scales for an African American population took place in 1997(Champion & Scott, 1997).Later, the Champion HBM scales were then tested among a variety of racial and ethnic groups and translated into several languages. Study participants included 100 first-generation Chinese American women(Wu & Yu, 2003), 656 from Turkey (Mikhail & Petro‐Nustas, 2001; Secginli & Nahcivan, 2004), 500 from Jordanian workers and students, and 264 females from south Korea. When the results of these investigations were combined, they validated the validity and reliability of translated HBM measures.

Since 1950, HBM has been one of the most often utilized conceptual frameworks in health behavior research to explain changes in health-related behaviors and serve as a basis for interventions. Intervention studies based on the HBM have revealed a significant improvement in BCS rates(Glanz, Rimer, & Viswanath, 2015) . Hence, the present study will consider the utility of the Champion HBM as a theoretical framework to understand Pakistani college teacher’s beliefs related to BC and to predict BCS.

## 1.5 Conceptual framework of study

Based on the literature review, this study conceptualized breast self-examination practice as a dependent variable. All independent variables, Individual factors (Sociodemographic & family history of breast cancer) and educational intervention, dependent variables which have effects on breast self-examination variable, that have been investigated in this study are demonstrated in Figure II.

Figure II: Conceptual framework of study

# Methodology

## 2.1 Study Location

The study will be conducted in Hyderabad district, located in the Sindh province of Southeast Pakistan. Hyderabad, positioned 175 kilometers north of Karachi, ranks as the second-largest city in Sindh and the eighth largest in Pakistan, with a population of 1,732,693 as per the 2017 census (Statistics, 2017). It serves as a significant hub connecting Greater Sindh with rural areas.

Ten colleges from Hyderabad District, with 686 teachers, are included. These colleges offer intermediate and degree programs. Their programs are in three main areas: Arts, Science, and Business (Commerce). The teachers at these colleges are very well educated. Most of them have master’s degrees in their subjects, and some even have PhDs. The minimum criteria for appointment are a master’s degree in the respective field. We will conduct several preliminary steps before starting the main study, a Cluster Randomized control trial. These include a pilot study and a pretest to make sure our research tools are accurate and effective. Additionally, a cross-sectional study will be carried out to gather initial data from the same group of people who will participate in the main study.

To ensure fairness, the ten colleges will be randomly divided into different groups. Each group will participate in a specific phase of the study. This random assignment helps to prevent bias and makes the results more reliable. Among these ten, six colleges are randomly selected for the Pilot & pretesting phase and the cross-sectional phase, leaving four colleges to include in the main study.

**Study Design:**

A single blinded, cluster randomized controlled trial (cRCT) will be carried out among female college teachers from government colleges in Hyderabad district. Colleges will serve as randomization units (clusters). The study will proceed through three phases:

**Baseline Data Collection:** Data will be collected from both control and intervention groups.

**Intervention Delivery**: The intervention will be administered to the intervention group during this phase.

**Follow-up Data Collection**: Follow-up data will be collected at two different post-intervention points: one month and three months later. The same questionnaire will be used for both control and intervention groups. The intervention module will be applied to the control group after the study's completion.

## 2.3 Study Population

The study population comprises full-time female college teachers aged 25-59 years, employed in government colleges in Hyderabad district.

## 2.4 Study Duration

The study will be conducted from September 2024 to January 2025.

## 2.5 Sampling Strategy

### 2.5.1 Sample Population

The sample will consist of college teachers aged 25-59 years from government colleges in Hyderabad district.

### A. Inclusion Criteria:

**For Colleges**

Willing to participate in the study.

**For Teachers**

Full-time faculty members appointed in government colleges in Hyderabad district.

Aged between 25-59 years.

willing to participate in the study.

### B. Exclusion Criteria:

**For Colleges**

Colleges are unwilling to participate despite meeting inclusion criteria.

Colleges that participated in initial phases of the study, pilot testing, or cross-sectional studies.

**For Teachers**

Pregnant or lactating teachers.

Teachers on sabbatical leave.

Diagnosed/recovered breast cancer patients.

Teachers are unwilling to participate in the study.

### 2.5.2 Sampling Frame

A comprehensive list of government colleges in Hyderabad district.

A complete list of teachers employed in government colleges in Hyderabad district, including their names and employment ID numbers obtained from the relevant department.

### 2.5.3 Sampling Unit

Each female teacher employed in government colleges.

## 2.6 Sample Size Calculation

The sample size was determined for a cluster randomized controlled trial with considerations for an average cluster size of 99, an intra-cluster correlation coefficient (ICC) of 0.02, and a 20% attrition rate. The final required sample size adjusted for eligibility proportion was approximately 114 teachers, with 57 participants allocated to each group (intervention and control). Refer Appendix II.

## 2.7 Sampling Methods

A cluster sampling method will be applied, four colleges will be included in CRCT.A thorough eligibility assessment will be conducted. This evaluation will consider factors including the availability of an adequate number of qualified teachers, infrastructure needs, and study participation readiness. In the proposed research study, the following process will be followed to select a representative sample and conduct randomization for the cluster randomized trial.

**Step 1:**

**Block randomization.**

In this study, we will use a block randomization approach to allocate the four participating colleges into two balanced blocks of block size 2, for our cluster randomized controlled trial. The allocation of colleges to the intervention and control groups will be based on the total number of teachers in each college, aiming to achieve balance and minimize potential biases. For instance, the allocation resulted in the following distribution:

Block 1: 200 teachers

- College A (112)
- College B (88)

Block 2: 198 teachers

- College C (117)
- College D (81)

Each block will be designed to have almost the same total number of teachers from different colleges, ensuring that the allocation will be as balanced as possible given the available information. This approach will be chosen to enhance the validity of our trial by reducing the impact of variations in college sizes on the study's outcomes.

**Step 2:**

**Randomize Within Blocks:**

- Each block will be randomly assigned to one of the treatment groups. For example, flip a coin or use a random number generator to determine whether Block 1 goes to the intervention or control group, and similarly for Block 2.

**Required sample size:** The Total sample size required for the second phase of study is 114, that is 57 participants in each arm.

**Step 3: Calculation of Proportions and allocated sample size for Each College:**

**Intervention Arm**

1. College A= (112) - 112/ 112+88*57=31
2. College B = (88)- 88/112+88*57=26

**Control Arm**

1. College C (117) - 117/117+81*57=34
2. College D (81) 81/117+81*57=23

**Step 4: Proportionate Stratified sampling:**

Proportionate stratified sampling from all colleges based on faculty will be performed once the assignment of colleges is done to the intervention and control group, e.g., 2 colleges in the intervention group.

To calculate the proportions for each faculty within the intervention group (57) for the two colleges (A & B), these steps can be followed.

Proportions will be calculated based on the number of teachers in each faculty at each college.

e.g.

**College A=Total number of teachers= 112**

Arts: (52 / 112) *31 =14 participants

Science: (49/ 112) * 31 = 14 participants

Commerce: (11/ 112) * 31 = 03 participants

**2. College B: Total number of teachers=88**

Arts: (40 / 88) * 26 = 12 participants

Science: (37 / 88) * 26 = 10 participants

Commerce: (11 / 88) * 26= 4 participants

Similarly stratified proportionate sampling will be done for the Colleges of Control group.

**Step 5 Random sampling:**

Participants will be selected randomly by random number generators, randomly selected participants will be invited for the study, exclusion and inclusion criteria will be discussed; written consent will be obtained from eligible participants, and baseline data will be collected.


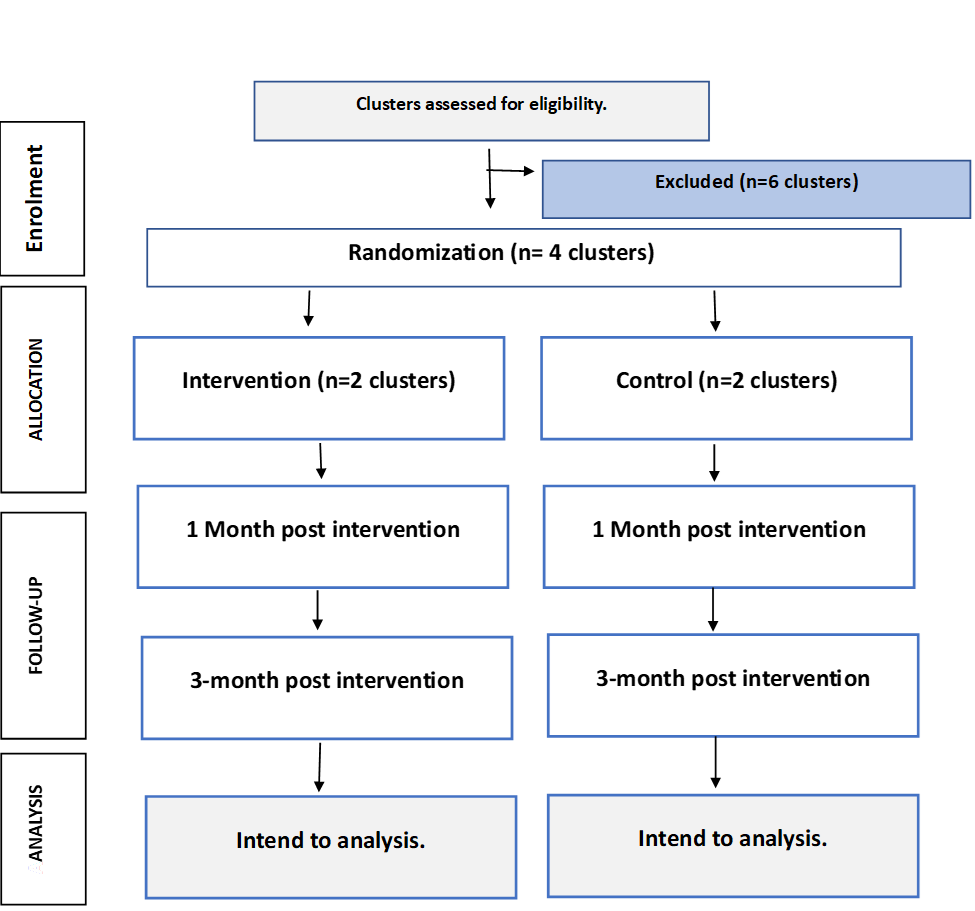


Figure III. CONSORT flow diagram of the study—adapted from Campbell (Campbell, Elbourne, & Altman, 2004)

## 2.8 Study outcomes

**2.8.1 Primary outcome**

The primary outcome for this study is the practice of Breast Self-Examination (BSE). This will be assessed based on the percentage of participants who consistently and effectively perform breast self-examinations. Participants will self-report on their BSE practices using a structured questionnaire. Regular BSE practice will be defined as performing the examination once a month, while any other response indicating irregular BSE will be categorized as non-practicing.

**2.8.2 Secondary outcome**

The secondary outcomes of this study encompass two domains: knowledge of breast cancer and breast self-examination (BSE), and beliefs related to breast cancer and BSE.

**Knowledge of Breast Cancer and BSE:** The study will assess participants' knowledge through a comprehensive self-report questionnaire. These items will cover various aspects including breast cancer risk factors, signs and symptoms, breast health awareness including BSE practices, and breast cancer screening techniques. Participants' understanding of these critical areas will be evaluated to gauge their baseline knowledge and any improvements post-intervention.

**Beliefs about Breast Cancer and BSE:** Beliefs related to breast cancer and BSE will be measured using the modified Urdu version of the Champions Health Belief Model Scale (CHBMS). This scale will be modified, translated and validated by the researchers for this study. These subscales include perceptions of the severity and seriousness of breast cancer, perceived benefits and barriers of performing BSE, confidence levels in performing BSE, and motivation towards adopting BSE practices. By using this scale, the study aims to capture participants' attitudes and beliefs regarding breast cancer prevention and early detection through self-examination.

These secondary outcomes are crucial in understanding how participants' knowledge and beliefs influence their behaviors related to breast cancer screening practices, thereby informing the effectiveness of the educational intervention designed for this study.

## 2.9 Study instrument

A self-administered questionnaire will be used, the Questionnaire includes four sections:

**Section I** comprises socioeconomic characteristics (age, marital status, teaching experience, religion, family income, education level, native language, area of residence, presence of health personnel in immediate family, personal history of benign diseases, family history of breast cancer).

**Section II**: It consists of questions based on,

- Risk factors of breast cancer
- Symptoms of breast cancer

- BCS methods
- BSE

Responses will be measured by utilizing the nominal scale of “True”, “False” and “I do not know”. Respondents will score one point for every correct answer and will score zero points for uncertain or wrong response. The maximum knowledge considered will be 36and the minimum score will be 0. Scores will later be calculated for analysis; higher scores will be associated with greater knowledge.

**Section III: Health Belief Model Scales**

The CHBMS Champion (1993) and Champion (1999) will be used after getting permission from authors, in the current study, for measuring beliefs related to BC. The CHBM scale based on 6 self-reported measured scales are perceived susceptibility to breast cancer (5 items), perceived severity of breast cancer (7 items), perceived benefits of BSE (6 items), perceived barriers for breast self-examination (6 items), confidence in one’s ability to perform Breast self-examination (11 items), health motivation (7 items),., All scale items will be measured with a 5 point Likert scale with the coding mentioned below.

- Strongly disagree One point
- Disagree Two points.
- Neutral Three points
- Agree Four points.
- Strongly agree Five points

Then scores will be summed up for analysis. Higher scores will represent greater perception linked with that construct (Champion, 1995). All HBM scales are associated positively with breast cancer screening behaviors, excluding the barrier scale, which is linked negatively. Researchers used Bloom's cut-off point to categorize the total beliefs scores into three categories:(Abdullahi et al., 2016; Yimer et al., 2014).

- **Negative attitude:** (scores representing 0-49%)
- **Neutral attitude:** (scores representing 50-79%)
- **Positive attitude:** (scores representing 80-100%)

**Section IV: Breast self-examination practice and frequency**

BSE, practice, and frequency of performing BSE test will be measured by utilizing a self-reported instrument.

**Translation of original CHMBS into Urdu language:**

To translate the CHBMS into Urdu for use among Pakistani females, will be started by forming a specialized translation team comprising bilingual experts proficient in both English and Urdu. This team will include linguists with expertise in medical terminology, public health professionals familiar with the CHBMS, and native Urdu speakers to ensure the translation's cultural relevance and accuracy. Initially, two independent translators will translate the CHBMS from English to Urdu, following guidelines to maintain consistency in terms and concepts. These translations will then be reviewed by the team to address any discrepancies.

Next, is the reconciliation and harmonization of the translations into a single coherent version. A committee, including the initial translators and additional experts, will review and resolve any differences to ensure the Urdu version accurately reflects the original content and is culturally appropriate for Pakistani females. Following this, the Urdu version will undergo back-translation, where two independent translators, not involved in the initial translation, will translate the Urdu version back into English. This process will help identify inconsistencies or errors, which will be addressed through further refinement of the Urdu translation.

To ensure the translation's comprehensibility and cultural relevance, the Urdu version will be pre-tested with a small group of Pakistani females. Cognitive interviews will be conducted to gather feedback on the clarity and cultural appropriateness of the translation, and adjustments will be made based on this feedback. After final revisions, Urdu CHBMS will undergo a final review by the translation team and additional experts to ensure accuracy and appropriateness.

## 2.10 Quality control of the questionnaire

**Validity**

**Face Validity**

To ensure the reliability and effectiveness of our questionnaire, face validity will be assessed by five experts from University Putra Malaysia. Face validity refers to the extent to which a test or questionnaire appears to measure what it is supposed to measure, based on subjective judgment. This type of validity is important as it will help establish the credibility of the instrument in the eyes of respondents and stakeholders. The experts will review the questionnaire for clarity, relevance, and comprehensiveness, providing critical feedback on the appropriateness of the questions and their alignment with the study objectives.

In addition to expert validation, feedback from a group of five participants will also be collected. This step will be crucial for understanding how the target population perceives the questionnaire. The participants' feedback will help identify any ambiguities or difficulties in understanding the questions, ensuring that the language and content are appropriate and comprehensible to the intended audience. This dual approach of involving both experts and participants in the validation process will help enhance the questionnaire's face validity, making it a more reliable tool for measuring knowledge, beliefs, and practices related to breast self-examination. By addressing potential issues identified during this phase, we will improve the questionnaire's overall quality and ensure that it will effectively capture the necessary data for our study.

**Content validity**

To ensure the robustness and relevance of our questionnaire, content validity will be rigorously assessed by a panel of experts from University Putra Malaysia (UPM) and Pakistan. Content validity involves evaluating whether the instrument will comprehensively cover the construct it aims to measure. This process will help ensure that all relevant aspects of the subject are included, and the items accurately reflect the intended content domain.

Each item in the questionnaire will be evaluated for content validity using the Content Validity Index (CVI). CVI is a widely used statistical measure that quantifies the degree of agreement among experts regarding the relevance of individual items. For our study, the Item-level Content Validity Index (I-CVI) will be calculated for each question. The I-CVI represents the proportion of experts who rate the item as either relevant or very relevant.

Items with an I-CVI above a predetermined threshold, such as 0.78 or 0.80, will be considered to have good content validity. This threshold will ensure a high level of agreement among the experts regarding the relevance and appropriateness of the items. Items that do not meet this criterion will be reviewed and revised based on the experts' feedback or removed from the questionnaire to maintain the overall quality and relevance of the instrument.

This thorough process of assessing both face and content validity will help ensure that our questionnaire is not only clear and understandable to participants but also comprehensive and relevant to the study's objectives. By incorporating expert feedback and adhering to rigorous validation criteria, we aim to create a reliable tool capable of accurately measuring knowledge, beliefs, and practices related to breast self-examination among college teachers in Hyderabad, Pakistan.

**Reliability**

The reliability of our measurement instrument will be assured through various strategies tailored to the study's context and informed by previous research. Specifically, for items designed to measure attitudes, we will assess reliability using Cronbach's alpha, with a threshold set at 0.70 or higher to ensure internal consistency. Corrections will be made based on test outcomes to enhance reliability. Furthermore, employing consistent data collectors who will assess research participants both at baseline and during follow-up will add an extra layer of assurance regarding the instrument's reliability.

The questionnaire to be utilized in this cluster randomized controlled trial (cRCT) will be a validated and reliable tool, rigorously tested for validity and reliability by the study researchers before its deployment.

## 2.11 Data collection

With the help of college coordinators, small meeting groups will be arranged in colleges. All the selected participants will be informed about the purpose and procedures of breast cancer awareness and BSE practices educational intervention trial by the researcher as well as the selection criteria will be explained.

After reading through the contents of the respondent information sheet, all participants who willingly show consent to be part of the study will be given a consent form to sign. Afterward, they will be requested to fill out a structured pre-tested baseline breast cancer knowledge, beliefs, and practice questionnaire in the English language. 1 month and 3 months post intervention, the same questionnaire will be administered to the intervention group and the control group respectively.

The same strategy will be followed in the control colleges, teachers will be selected randomly, but the purpose and procedure about the intervention will not be discussed, only the data collection procedure will be discussed with them. After getting written consent from them, they will be requested to fill in the questionnaire at the baseline, 1-month post-intervention (intervention delivered to intervention group), and three months post-intervention.

## 2.12 Delivery of intervention module and participation

1. **Knowledge-based awareness session.**

After the collection of baseline data, a 60-minute one-day session of the educational program on breast cancer awareness and screening will be delivered to each intervention college by the study researcher with a group of 20 to 25 participants. Before implementing the intervention, researchers will ensure that all the participants must have a mobile phone to receive the reminder SMS and to watch the BSE video clip. The procedure description will be as follows,

One hour presentation along with a short video of 5 minutes duration about BSE performance will be presented, which will consist of general structure as well as functioning of normal breast, awareness on breast health, basic knowledge on breast cancer, and education on various screening methods will be provided by the researcher.

1. **Skill based training session.**

Followed by the training session, in which a breast model made up of silicon having multiple lumps on it will be used for demonstration of BSE method, during this section, participants will learn the palpation technique, the search strategy, and signs of BC that are important for respondents to know. Later, the participants will be asked to perform BSE on the model and look for abnormalities.

1. **Take home messages for participants.**

A leaflet with the same information that was provided in the session will be given to every participant when the educational session ends.

A short film about BSE performance will be sent to the participants via WhatsApp, and a BC logo wall hanging will also be provided to be hung in the participants ‘room. Such materials will be believed to remind participants about BCS practice and monthly BSE performance. A short WhatsApp reminder message will be sent to all participants of the intervention group for the period of three months.

1. **Souvenirs as a reminder for breast self-examination**

Some souvenirs will be given to the participants, like a key chain, a writing pen, and a note pad with the breast cancer logo and a message “**know your risk**” get screening timely! To remind them of breast self-examination.

**For Control Group**

Control cohort receives the same intervention material along with practical on the performance of BSE right after the collection of the second post-intervention follow-up response from both groups.

topics as the baseline assessment to track changes in knowledge, beliefs, and practices

## 2.13 Retention of Participants

To overcome the attrition, various participants will be followed to retain the participants till the end, detailed information regarding the participant's contact will be acquired, like enrolment number, contact number, and email address will be obtained from the participants, and the researcher will make sure nobody else can have access to them. The researcher will share her contact number and email address for any correspondence associated with the BSE examination.

## 2.14 Evaluation of the Educational Intervention

For both groups, evaluation follow-up will be accomplished in one month, and a month follow up. Content of the follow-up questionnaire assessment will be the same as of the baseline questionnaire, except for section one, which will be omitted from the follow-up assessment. The differences among both groups for breast self-examination practice, knowledge and beliefs associated with breast cancer, will be assessed by statistical tests.

## 2.15 Development and validation of intervention module

Integrating guidelines from the American Cancer Society (ACS) into our intervention module for breast cancer screening will represent a pivotal step in ensuring credibility and effectiveness. The ACS, renowned for its expertise in cancer research and prevention, provides evidence-based recommendations crafted by leading breast cancer experts. This integration will not only bolster the module's validity but also align our efforts with globally recognized standards for breast cancer screening and prevention (Rock et al., 2020).

The ACS guidelines will serve as a benchmark for promoting consistency and best practices in our intervention. Regular updates based on the latest scientific research will ensure that our module incorporates current knowledge and advancements in the field, thereby enhancing its relevance and reliability (R. A. Smith & K. C. Oeffinger, 2020). By adhering to these guidelines, our intervention will not only educate but also empower participants with accurate, up-to-date information critical for making informed decisions about breast cancer screening (Desreux, 2018).

Furthermore, grounding our intervention in the Health Belief Model (HBM) will provide a robust theoretical framework for understanding and influencing health behaviors related to breast cancer screening. The HBM emphasizes individual perceptions of susceptibility to breast cancer, the seriousness of the disease, and the perceived benefits and barriers to screening (Rezano et al., 2022). By addressing these factors, our intervention aims to modify beliefs and attitudes, thereby fostering a proactive approach to breast cancer prevention among teachers in our study.

By addressing these factors, our intervention aims to modify beliefs and attitudes, thereby fostering a proactive approach to breast cancer prevention among teachers in our study. The intervention protocol will include a comprehensive educational module delivered through multimedia presentations, skill-based training sessions, informative leaflets, and engaging videos on breast health and self-examination. Additionally, practical aids such as breast cancer logo wall hangings and reminder messages via WhatsApp will aim to reinforce the importance of regular self-examination (Ştefănuţ & Vintilă, 2022). Participants will also receive small souvenirs featuring breast cancer awareness messages, serving as daily reminders to prioritize their health.

The development of the educational module will undergo rigorous validation processes, including content validity checks by experts from the Community Health Department. Their feedback will ensure that the module effectively conveys essential information and addresses potential cultural sensitivities and barriers to screening (Pucha, 2022).

In summary, by integrating ACS guidelines and leveraging the HBM, our intervention module aims to educate and empower participants to take proactive steps towards breast cancer screening. Through a robust educational approach and rigorous validation processes, we strive to enhance awareness, promote early detection, and ultimately reduce the burden of late-stage breast cancer diagnoses in our community

## 2.16 Operational Definitions of Variables

**13.15.1 Dependent Variables**

**1. Practice of Breast Self-Examination (BSE)**

- **Frequency of BSE:** Participants will be categorized as practicing BSE regularly (once a month or more) or irregularly (occasionally or less).
- **Performance of BSE:** Participants will be assessed on their knowledge of how to conduct BSE through a series of questions.

**2. Secondary Dependent Variables**

- **Knowledge of Breast Cancer:** Measured by summing scores on a knowledge assessment. Scoring will be categorized as, Low knowledge for scores (0-49%), Moderate knowledge for scores (50-79%) and High knowledge for scores (80-100%)(Abdullahi et al., 2016; Yimer et al., 2014).
- **Knowledge of Breast Self-Examination:** Measured similarly to knowledge of breast cancer, with the same scoring criteria.

**Health Beliefs Associated with BC & BSE:** Assessed using a 5-point Likert scale to measure agreement with statements related to Health Belief Model constructs. Higher scores indicate stronger agreement with these beliefs. Researchers used Bloom's cut-off point to categorize the total beliefs scores into three categories:(Abdullahi et al., 2016; Yimer et al., 2014).

Negative attitude: Scores ranging from 32 to Therefore, the corrected cut-off points for your 32-statement Likert scale are:

- **Negative attitude:** 32 to 78 (scores representing 0-49%)
- **Neutral attitude:** 79 to 126 (scores representing 50-79%)
- **Positive attitude:** 127 and above (scores representing 80-100%)

**13.15.2 Independent Variables**

**a. Educational Intervention on Breast Cancer and BSE** The intervention group will receive a PowerPoint presentation, hands-on training using a breast model, and additional materials (leaflet, short film, wall hanging, SMS reminders). The control group will receive no intervention.

**b. Sociodemographic Characteristics**

- **Age:** Calculated as the difference between birth year and the study year. Participants must be 25 years old or older.
- **Years of Teaching Experience:** Self-reported by participants.
- **Monthly Income:** Categorized into low, middle, and high income based on World Bank income levels
- **Education Level:** Highest educational qualification achieved (PhD, Master's, Bachelor's).
- **Marital Status:** Self-reported as single, married, divorced/separated, or widowed.
- **Religion:** Self-reported as Islam, Christianity, Hinduism, or other.
- **Native Language:** Self-reported from a list of options.
- **Presence of Female Doctor/Health Personnel in Immediate Family:** Yes or no.
- **Area of Residence:** Categorized as rural or urban.
- **Family History of Breast Cancer:** Self-reported as yes or no.
- **Personal History of Benign Breast Diseases:** Self-reported as yes or no.

## Data Summarizing and Analysis

The statistical analysis for this study will be conducted using IBM SPSS Statistics software, version 26.0. Before performing any statistical tests, continuous variables will be evaluated for their distribution characteristics. Variables that display a normal distribution will be summarized using descriptive statistics such as mean and standard deviation. In contrast, continuous variables that do not follow a normal distribution will be described using the median and interquartile range (IQR).

For categorical or dichotomous data, descriptive statistics will include percentages, counts, or proportions to effectively summarize the data. Statistical comparisons between categorical data from the intervention and control groups will be conducted using Chi-square tests or Fisher's exact tests, as appropriate. Differences in means of normally distributed continuous variables between these groups will be assessed using independent-samples t-tests. Meanwhile, non-normally distributed continuous data will be compared using the Mann-Whitney U test.

Given the clustered nature of the study design, Generalized Estimating Equations (GEE) will be employed for statistical analysis. GEE is robust for analyzing data that exhibit correlation within clusters (e.g., colleges and teachers within colleges). It will enable the assessment of group effects (intervention vs. control), time effects (pre- vs. post-intervention), and their interaction effect (group*time) over the study period.

We will analyze all the data collected from both the intervention and control groups, even if participants drop out. This approach is called Intention-to-Treat (ITT). If someone stops participating before the study ends, we will use their last recorded information to fill in any missing data. This method helps ensure that our results are accurate and unbiased, even when people leave the study early.

## **2.17 Results dissemination**

Study findings will be communicated to participants, the community, and pertinent stakeholders in time. Clear and comprehensible information about the results, their implications will be provided and any recommendations for practice will be provided if necessary

## 2.18 Permissions and Ethical clearance

This study obtained necessary permission and ethical clearance from the College Education Department, letter no. **DCEHRH- 2023-24/423** Government of Sindh, Pakistan, and applied for ethical approval from the local Institutional Review Committee of the Mekran Medical College, for ethical clearance. Written informed consent from study participants will be acquired.

## References

Abdullahi, A., Hassan, A., Kadarman, N., Saleh, A., Baraya, Y. u. S. a., & Lua, P. L. (2016). Food safety knowledge, attitude, and practice toward compliance with abattoir laws among the abattoir workers in Malaysia. *International journal of general medicine*, 79-87.

ABDULRAHMAN, S. N. (2020). *EDUCATIONAL INTERVENTION IN BREAST CANCER SCREENING UPTAKE, KNOWLEDGE AND BELIEFS AMONG YEMENI FEMALE SCHOOL TEACHERS IN THE KLANG VALLEY, MALAYSIA* University Putra Malaysia]. Malaysia.

Agha, N., & Rind, R. D. (2021). Beliefs and perceptions about breast cancer among the people living in rural and less privileged areas in Sindh, Pakistan. *Health Education*.

Almutari, N., & Orji, R. (2021). Culture and Health Belief Model: Exploring the determinants of physical activity among Saudi adults and the moderating effects of age and gender. Proceedings of the 29th ACM Conference on User Modeling, Adaptation and Personalization,

Champion, V., Skinner, C. S., & Menon, U. (2005). Development of a self‐efficacy scale for mammography. *Research in nursing & health*, *28*(4), 329-336.

Champion, V. L. (1999). Revised susceptibility, benefits, and barriers scale for mammography screening. *Research in nursing & health*, *22*(4), 341-348.

Champion, V. L., & Scott, C. R. (1997). Reliability and validity of breast cancer screening belief scales in African American women. *Nursing research*, *46*(6), 331-337.

Charan, J., & Biswas, T. (2013). How to calculate sample size for different study designs in medical research? *Indian journal of psychological medicine*, *35*(2), 121-126.

Fahad Ullah, M. (2019). Breast cancer: current perspectives on the disease status. *Breast Cancer Metastasis and Drug Resistance: Challenges and Progress*, 51-64.

Fitzgerald, R. C., Antoniou, A. C., Fruk, L., & Rosenfeld, N. (2022). The future of early cancer detection. *Nature medicine*, *28*(4), 666-677.

Glanz, K., Rimer, B. K., & Viswanath, K. (2015). *Health behavior: Theory, research, and practice*. John Wiley & Sons.

Government of pakistan , f. d. (2022). *Pakistan Economic Survey 2021-22*. <https://www.finance.gov.pk/survey/chapter_22/PES10-EDUCATION.pdf>

Hayden, J. (2022). *Introduction to health behavior theory*. Jones & Bartlett Learning.

Htay, M. N. N., Schliemann, D., Dahlui, M., Cardwell, C. R., Loh, S. Y., Tamin, N. S. B. I., Somasundaram, S., Champion, V., Donnelly, M., & Su, T. T. (2021). Validation of the champion health belief model scale for an investigation of breast cancer screening behaviour in Malaysia. *International Journal of Environmental Research and Public Health*, *18*(17), 9311.

Javaeed, A., Rasheed, I., & Shafqat, F. (2021). Knowledge, attitude, and practice of breast self-examination among female undergraduate medical students in Poonch Medical College, Azad Kashmir. *JPMA. The Journal of the Pakistan Medical Association*, *71*(2 (A)), 524-527.

Mikhail, B. I., & Petro‐Nustas, W. I. (2001). Transcultural adaptation of Champion's health belief model scales. *Journal of Nursing Scholarship*, *33*(2), 159-165.

Miller, K. D., Fidler‐Benaoudia, M., Keegan, T. H., Hipp, H. S., Jemal, A., & Siegel, R. L. (2020). Cancer statistics for adolescents and young adults, 2020. *CA: a cancer journal for clinicians*, *70*(6), 443-459.

Oguta, M. A., Ayodo, G., Humwa, F., & Awandu, S. S. (2022). Knowledge and Practice of Breast Self-Examination for Early Detection of Breast Cancer Among Young Women Attending Maternal Health Clinic at JOOTRH, Kisumu County.

Ralph.J.Clemente, L. F. S., Richard A.Crosby. (2021). *Health behaviour theory for public health.* (2nd ed.). ‎Jones & Bartlett Learning.

Ramdas, Y., Benn, C.-A., & van Heerden, M. (2020). First intraoperative radiation therapy center in Africa: First 2 years in operation, including COVID-19 experiences. *JCO global oncology*, *6*, 1696-1703.

Richards, S., Bankhead, C., Peters, T., Austoker, J., Hobbs, F., Brown, J., Tydeman, C., Roberts, L., Formby, J., & Redman, V. (2001). Cluster randomised controlled trial comparing the effectiveness and cost-effectiveness of two primary care interventions aimed at improving attendance for breast screening. *Journal of Medical Screening*, *8*(2), 91-98.

Saeed, S., Asim, M., & Sohail, M. M. (2021). Fears and barriers: problems in breast cancer diagnosis and treatment in Pakistan. *BMC Women's Health*, *21*, 1-10.

Secginli, S., & Nahcivan, N. O. (2004). Reliability and validity of the breast cancer screening belief scale among Turkish women. *Cancer Nursing*, *27*(4), 287-294.

Shahani, M. P., Ahmer, A., Siyal, F. J., Soomro, S. H., Humayun, A., Shaikh, A. A., Abbas, J., Shahani, Z., Saleem, R., & Unar, A. A. (2020). Breast self examination: knowledge and practice of young female students towards breast self examination and breast cancer. *J Pharm Res İnt*, *32*(24), 93-100.

Sobri, F. B., Bachtiar, A., Panigoro, S. S., Ayuningtyas, D., Gustada, H., Yuswar, P. W., Nur, A. A., Putri, R. C. R. A., & Widihidayati, A. D. (2021). Factors affecting delayed presentation and diagnosis of breast Cancer in Asian developing countries women: a systematic review. *Asian Pacific Journal of Cancer Prevention: APJCP*, *22*(10), 3081.

Wu, T.-Y., & Yu, M.-Y. (2003). Reliability and validity of the mammography screening beliefs questionnaire among Chinese American women. *Cancer nursing*, *26*(2), 131-142.

Yimer, M., Abera, B., Mulu, W., & Bezabih, B. (2014). Knowledge, attitude and practices of high risk populations on louse-borne relapsing fever in Bahir Dar city, north-west Ethiopia. *Science Journal of Public Health*, *2*(1), 15-22.

### Appendix I. Study Gantt Chart


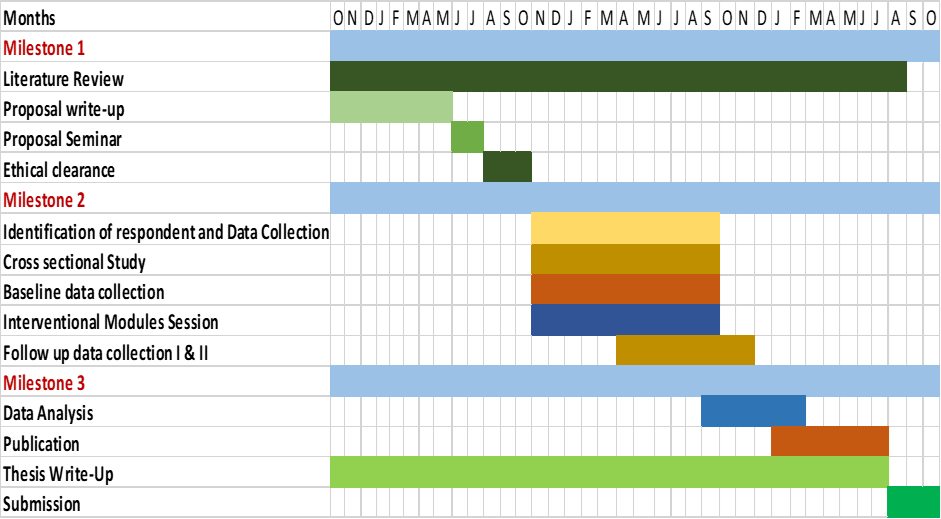


### Appendix II. Sample size calculations

Phase II(CRCT)

To determine a significant association between the respondent’s characteristics, knowledge, beliefs, and practices on breast self-examination among college teachers.

Sample size calculation will be done by applying formula two proportion/two sample for t testing hypothesis and observing expected differences in proportions of BSE practice between two groups.

n = [(Z_ı - α)√(2P̅(1 - P̅)) + (Z_ı - β)√(Pı(1 - Pı) + P2(1 - P2))] / (Pı - P2)²

Table 3.3: Information of sample size derivation

| **Attribute** | **Assumptions** |
| --- | --- |
| **Z** | Cluster Randomized Controlled Trial |
| **Z(1-α)** | Zα= 1.96; p-value= 0.05; therefore, power desired is 0.95 |
| **Z(1−β)** | Zβ=0.84; β=0.20; 0.80 power was desired. (James 2001) |
| **P1** | P1=85.6% Prevalence of regular breast self-examination practice in intervention group 06 months post intervention(ABDULRAHMAN, 2020) |
| **P2** | P2=26%, Prevalence of regular breast self-examination practice in control group 06 months post intervention(ABDULRAHMAN, 2020) |

Sample size n= **14** calculated by openEpi(Charan & Biswas, 2013) 2 groups 14* 2 = 28

28 x [1 + (m-1) ICC)

Where: m=average cluster size,

ICC-class correlation coefficient m

ICC =0.02: intra-cluster correlation coefficient based on Cluster randomised controlled trial comparing the effectiveness and cost-effectiveness of two primary care interventions aimed at improving attendance for breast screening (Richards et al., 2001),

m= 99 (average cluster size calculated)

n​=28×[1+(m−1) ×ICC]

Given that average cluster size =99

ICC=0.02:

n=28× [1+(99−1) ×0.02]

n=28× [1+98×0.02]

n=28× [1+1.96]

n=28×2.96

n≈82.88

Rounding up to the nearest whole number, the final adjusted sample size without considering attrition and eligibility adjustments would be approximately 83.

To adjust for a 20% attrition rate, we need to increase the sample size by dividing it by the complement of the attrition rate, which is 1-0.2 = 0.8. So:

83 / 0.8 = 103

To adjust for the eligibility proportion of 90%, we need to further increase the sample size by dividing it by the eligibility proportion. So:

103 / 0.9 = 114

The final required sample size with attrition and eligibility adjustments is approximately 114 teachers.

114/2 =57

57 participants in each group.

### Appendix III. RESPONDENT’S INFORMATION SHEET AND CONSENT & INSTRUMENT

1. **STUDY TITLE**

Effectiveness of a health belief model-based educational intervention on knowledge, beliefs, and practices of breast self-examination among college teachers in Hyderabad, Pakistan.

1. **INTRODUCTION**

Breast cancer is the most frequently occurring cancer type among females worldwide. Pakistan is one of the Asian countries with a higher prevalence and immature mortality due to breast cancer and ranks top among all the other cancer types in prevalence and death. In Pakistan, 82% of patients are diagnosed late and among them, 52% are diagnosed at an advanced stage with very less or almost no survival chances. Breast cancer greatly impacts the physiological and psychological well-being of patients; furthermore, available cancer treatment also exerts severe health consequences, emotionally and physically. However effective population-based screening strategies can make it possible to detect cancer at the very initial stage among asymptomatic females. Thereby reducing the burden of disease both for patients and for the health-providing system. Breast self-examination is the technique by utilizing that females can detect any abnormal tumor in their breast at home without the assistance of any health care provider or any equipment. Breast self-examination is a self-awareness strategy that assists individuals in recognizing the normal structure and shape of their breasts. It empowers them to promptly identify any abnormal signs or symptoms associated with breast cancer. Breast self-examination is generally practiced by females in developed countries and included in their routine however in Pakistan earlier research reported that it is very less practiced by Pakistani females and intervention and awareness sessions are recommended by early researchers to improve BSE practices. The current study aims to determine the impact of a health belief model-based educational intervention on knowledge, beliefs, and practices of breast self-examination among college teachers in Hyderabad, Pakistan.

1. **WHAT WILL YOU HAVE TO DO?**

You are required to sign the informed consent form to show your willingness to be part of this study. If you are willing to participate in the current study, your cooperation is required to:

1. Fill out the questionnaire at three-time points.
2. This questionnaire is composed of four sections (Socio-demographic characteristics, risk factors of breast cancer, knowledge and beliefs about breast cancer, and BSE practices).
3. **WHO SHOULD NOT PARTICIPATE IN STUDY?**

Teachers who have been diagnosed with breast cancer. Teachers who are absent on the day of intervention and who are planning to go on long leave for the next three months and teachers who are retiring during the study period cannot take part in the study.

1. **WHAT WILL BE THE BENEFITS OF THE STUDY?**
2. **TO YOU AS THE SUBJECT**

As a subject, the current interventional study will make you more knowledgeable about breast cancer and develop stronger opinions regarding its early diagnosis. The study's findings may change how you personally feel about breast cancer screening and inspire you to conduct breast self-examination in the right way and at the right time.

1. **TO THE INVESTIGATOR**

Your involvement in this study will benefit the field of community health by allowing for the provision of better information, safety measures, and preventative measures to teach women about breast cancer early detection. Health educators may be helped by the provision of baseline data on the degree of breast cancer awareness among Pakistani women in promoting prevention programs.

1. **WHAT ARE THE POSSIBLE RISKS?**

It is expected that there will be no risk of any kind to study participants and organizations while conducting this study.

1. **WILL THE INFORMATION THAT YOU PROVIDE AND YOUR IDENTITY REMAIN CONFIDENTIAL?**

Results of current research will be reported collectively, without any kind of reference of the college or participants of the study, hence the details of participants will be kept confidential, and results will be used only for research purposes.

Please sign here if you have read and understood the contents of this page.

**CONSENT**

I …………………………………… Identity Card No. ……………………………

address……………………………………………………………………………………

…………………...……………………………………………...............………. I hereby freely consent to participate in the research described above (clinical/drug trial/video recording/focus group/questionnaire-based interview).

According to the Respondent's Information Sheet, I have been informed about the research methodology, potential drawbacks, and issues. I am aware that I have the option to leave this research at any moment and without providing any explanation. I know that the information I disclose regarding my identity will be kept private and secret, and this study is confidential.

Signature …….………………………… Signature …….………………………….

(Respondent) (Witness)

Date ...............................................................

Name………………………….

I/C No. ………………………………….

I confirm that I have explained to the respondent the nature and purpose of the above-mentioned research.

Date …….………………………… Signature …….………………………….

(Researcher)

**SECTION II: KNOWLEDGE**

**2.1 KNOWLEDGE OF BREAST CANCER RISK FACTOR**

|  |  | YES | NO | I DON’T KNOW | Rating |
| --- | --- | --- | --- | --- | --- |
| BCRF1 | Being obese or having dense breast tissue is a known risk factor for breast cancer. |  |  |  |  |
| BCRF 2 | The risk of breast cancer increases with age. |  |  |  |  |
| BCRF 3 | A family history of breast cancer elevates the chances of developing breast cancer. |  |  |  |  |
| BCRF 4 | Abnormal genes inherited from parents can increase the risk of breast cancer. |  |  |  |  |
| BCRF 5 | A personal history of benign(noncancerous)diseases increases the chances of breast cancer. |  |  |  |  |
| BCRF 6 | First childbirth at a later age after 30 increases the likelihood of developing breast cancer. |  |  |  |  |
| BCRF 7 | Prolonged breastfeeding for one year or more may lower the risk of breast cancer in women. |  |  |  |  |
| BCRF 8 | Delivering to several children more than five or six reduces the chances of breast cancer |  |  |  |  |
| BCRF 9 | Taking birth control pills and hormones increases the risk of breast cancer. |  |  |  |  |
| BCRF 10 | After menopause, being obese increases the risk of breast cancer. |  |  |  |  |
| BCRF 11 | The late onset of menopause after 55 is associated with a higher risk of breast cancer. |  |  |  |  |
| BCRF 12 | Early menarche increases the risk of breast cancer. |  |  |  |  |
| BCRF 13 | Does radiation therapy in the chest area increase the chances of breast cancer? |  |  |  |  |

**2.2 KNOWLEDGE OF BREAST CANCER SYMPTOMS**

|  |  | YES | NO | I don’t know | Rating |
| --- | --- | --- | --- | --- | --- |
| BCS1 | Are changes in the size or shape of the breast considered possible symptoms of breast cancer? |  |  |  |  |
| BCS2 | Is nipple discharge, other than breast milk, a potential symptom of breast cancer? |  |  |  |  |
| BCS3 | Are changes in the skin texture of the breast, such as redness or dimpling, indicative of breast cancer symptoms? |  |  |  |  |
| BCS4 | A painless lump in the breast is a sign of breast cancer. |  |  |  |  |
| BCS5 | Do breast cancer symptoms sometimes include unexplained pain or tenderness in the breast? |  |  |  |  |
| BCS6 | Are persistent breast or nipple itching a potential symptom of breast cancer? |  |  |  |  |
| BCS7 | Swelling and pain in the armpit region is a sign of breast cancer. |  |  |  |  |
| BCS8 | Change in shape and size of the nipple is a sign of breast cancer. |  |  |  |  |

**2.3 KNOWLEDGE OF BREAST CANCER SCREENING**

|  |  | YES | NO | I don’t know | Rating |
| --- | --- | --- | --- | --- | --- |
| BCS1 | Are you aware of different breast cancer screening methods like breast self-examination, clinical breast examination or mammography? |  |  |  |  |
| BCS2 | Early detection is the only way to get effective treatment? |  |  |  |  |
| BCS3 | A breast examination by a healthcare provider is an important step in the early detection of breast cancer |  |  |  |  |
| BCS4 | Mammography can identify breast cancer in its early stages. |  |  |  |  |

**2.4 KNOWLEDGE OF BREAST SELF EXAMINATION**

|  |  | Yes | No | I don’t know | Rating |
| --- | --- | --- | --- | --- | --- |
| BSE1 | Breast self-examination is a way for women to become aware of their breasts. |  |  |  |  |
| BSE2 | The right age to begin practicing breast self-examination is 20 years of age. |  |  |  |  |
| BSE3 | Breast self-examination can be performed while standing and lying down on the bed. |  |  |  |  |
| BSE4 | Breast self-examination should be performed once after period of three months. |  |  |  |  |
| BSE5 | The best time to perform breast self-examination is after the 7^th^ day of the menstrual cycle. |  |  |  |  |
| BSE6 | While performing breast self-examination during the shower, the individual should raise one arm and gently move the fingers on every part of the breast. |  |  |  |  |
| BSE7 | Menopausal women can perform breast self-examination at a particular date every month. |  |  |  |  |
| BSE8 | Both breasts should be examined in the same way to notice any change. |  |  |  |  |
| BSE9 | During breast self-examination, you should check for any skin thickening or lump in the breast. |  |  |  |  |
| BSE 10 | Breast self-examination should involve checking for lumps in the underarm region. |  |  |  |  |
| BSE11 | Performing breast self-examination while lying down, fingers flat press gently in small circles starting from the topmost corner of the breast. |  |  |  |  |

**SECTION III- BELIEFS (HBM scale)**

This section contained closed-ended questions which will be measure using a 5-point Likert scale statements ranging from (5) strongly disagree (4) disagree, (3) not sure, (2) agree, (1) strongly agree. Respectively.

|  |  | Strongly Agree | Agree | Neutral | Disagree | Strongly disagree |
| --- | --- | --- | --- | --- | --- | --- |
| SBC 1 | It is extremely likely I will get breast cancer in the future. |  |  |  |  |  |
| SBC2 | I feel I will get breast cancer in the future. |  |  |  |  |  |
| SBC3 | There is a good possibility I will get breast cancer in the next 10 years. |  |  |  |  |  |
| SBC4 | My chances of getting breast cancer are higher. |  |  |  |  |  |
| SBC5 | I am more likely than the average woman to get breast cancer. |  |  |  |  |  |

**3.1 SUSCEPTIBILITY OF BREAST CANCER**

**3.2 SERIOUSNESS OF BREAST CANCER**

|  |  | Strongly Agree | Agree | Neutral | Disagree | Strongly disagree |
| --- | --- | --- | --- | --- | --- | --- |
| SNBC 1 | The thought of breast cancer scares me. |  |  |  |  |  |
| SNBC 2 | When I think of breast cancer my heart beats faster. |  |  |  |  |  |
| SNBC 3 | I am afraid to think about breast cancer. |  |  |  |  |  |
| SNBC 4 | Problems I would experience with breast cancer would last a long time. |  |  |  |  |  |
| SNBC 5 | Breast cancer would threaten my relationship with my husband. |  |  |  |  |  |
| SNBC 6 | If I had breast cancer my whole life would change. |  |  |  |  |  |
| SNBC7 | If I developed breast cancer, I would not live longer than 5 years. |  |  |  |  |  |

**3.3 BENEFITS OF BSE**

| BBSE 1 | When I do breast self-examination, I feel good about myself. | Strongly agree | Agree | Neutral | Disagree | Strongly disagree |
| --- | --- | --- | --- | --- | --- | --- |
| BBSE 2 | When I complete monthly breast self-examination, I don’t worry as much about breast cancer. |  |  |  |  |  |
| BBSE 3 | Completing breast self-examination each month will allow me to find lumps early. |  |  |  |  |  |
| BBSE 4 | If I complete breast self-examination monthly during the next year, I will decrease my chance of dying from breast cancer. |  |  |  |  |  |
| BBSE 5 | If I complete breast self-examination monthly, I will decrease my chance of requiring radical or disfiguring surgery if breast cancer occurs. |  |  |  |  |  |
| BBSE 6 | If I complete monthly breast self-examination, it will help me to find a lump, which might be cancer before it is detected by a doctor or nurse. |  |  |  |  |  |

**3.4 BARRIERS OF BSE**

|  |  | Strongly agree | Agree | Neutral | Disagree | Strongly  disagree |
| --- | --- | --- | --- | --- | --- | --- |
| BRBSE 1 | I feel funny doing breast self-examination. |  |  |  |  |  |
| BRBSE 2 | Doing a breast self-examination during the next year will make me worry about breast cancer. |  |  |  |  |  |
| BRBSE 3 | Breast self-examination will be embarrassing to me. |  |  |  |  |  |
| BRBSE5 | Doing breast self-examination will be unpleasant. |  |  |  |  |  |
| BRBSE 6 | I don’t have enough privacy to do breast self-examination. |  |  |  |  |  |

**3.5 CONFIDENCE TO PERFORM BSE**

|  |  | Strongly agree | Agree | Neutral | Disagree | Strongly disagree |
| --- | --- | --- | --- | --- | --- | --- |
| CBSE 1 | I know how to perform breast self-examination. |  |  |  |  |  |
| CBSE 2 | I am confident I can perform breast self-examination correctly. |  |  |  |  |  |
| CBSE 3 | If I were to develop breast cancer, I would be able to find a lump by performing breast self-examination |  |  |  |  |  |
| CBSE 4 | I can find a breast lump if I  practice breast self-examination alone. |  |  |  |  |  |
| CBSE 5 | I can find a breast lump the size of a 5-cent coin. |  |  |  |  |  |
| CBSE 6 | I can find a breast lump the size of a 10-cent coin. |  |  |  |  |  |
| CBSE 7 | I can find a breast lump that is the size of a pea. |  |  |  |  |  |
| CBSE 8 | I am sure of the steps to follow for doing a breast self-examination. |  |  |  |  |  |
| CBSE 9 | I can identify normal and abnormal breast tissue when I do breast self-examination. |  |  |  |  |  |
| CBSE 10 | When looking in the mirror, I can recognize abnormal changes in my breast. |  |  |  |  |  |
| CBSE 11 | I can use the correct part of my fingers when I examine my breasts. |  |  |  |  |  |

**3.6 MOTIVATION**

|  |  | Strongly agree | Agree | Neutral | Disagree | Strongly disagree |
| --- | --- | --- | --- | --- | --- | --- |
| M1 | I want to discover health problems early. |  |  |  |  |  |
| M2 | Maintaining good health is extremely important to me. |  |  |  |  |  |
|  |  |  |  |  |  |  |
| M3 | I search for new information to improve my health. |  |  |  |  |  |
| M4 | I feel it is important to carry out activities to improve my health. |  |  |  |  |  |
| M5 | I eat well-balanced meals. |  |  |  |  |  |
| M6 | I exercise at least 3 times a week. |  |  |  |  |  |
| M7 | I have regular health check-ups even when I am not sick. |  |  |  |  |  |

**SECTION IV: PRACTICE**

**BSE Practice**

1. Do you practice BSE regularly to check for any changes or abnormalities in your breasts?

- Yes
- No

2. Which best describes the frequency of your Breast Self-Examinations (BSE)?

- Never
- Once in a month
- Once in a three-month period.
- Once in a six-month period.
